# Supplementary material for: Effect of relative income price on smoking initiation among adolescents in Ghana: evidence from pseudo-longitudinal data
Source: BMJ Open. 2022 Mar 16;12(3):e054367. doi: 10.1136/bmjopen-2021-054367 (PMC8928287; doi:10.1136/bmjopen-2021-054367)
Supplement: Author's manuscript [file bmjopen-2021-054367.draft_revisions.pdf]

BMJ Open is committed to open peer review. As part of this commitment we make the peer review history of every article we publish publicly available.

When an article is published we post the peer reviewers' comments and the authors' responses online. We also post the versions of the paper that were used during peer review. These are the versions that the peer review comments apply to.

The versions of the paper that follow are the versions that were submitted during the peer review process. They are not the versions of record or the final published versions. They should not be cited or distributed as the published version of this manuscript.

BMJ Open is an open access journal and the full, final, typeset and author-corrected version of record of the manuscript is available on our site with no access controls, subscription charges or pay-per-view fees (http://bmjopen.bmj.com).

If you have any questions on BMJ Open's open peer review process please email info.bmjopen@bmj.com

BMJ Open

The association between relative income price and smoking initiation among adolescents in Ghana

Journal:	*BMJ Open*
Manuscript ID	bmjopen-2021-054367
Article Type:	Original research
Date Submitted by the Author:	12-Jun-2021
Complete List of Authors:	Boachie, Micheal Kofi; University of Health and Allied Sciences, Health Policy Management<br>Immurana, Mustapha; University of Health and Allied Sciences, Institute of Health Research<br>Tingum, Ernest; University of Cape Town, Economics<br>Mdege, Noreen; University of York, Health Sciences<br>Ross, Hana; University of Cape Town, School of Economics
Keywords:	HEALTH ECONOMICS, PUBLIC HEALTH, SOCIAL MEDICINE

SCHOLARONE™
Manuscripts

I, the Submitting Author has the right to grant and does grant on behalf of all authors of the Work (as defined in the below author licence), an exclusive licence and/or a non-exclusive licence for contributions from authors who are: i) UK Crown employees; ii) where BMJ has agreed a CC-BY licence shall apply, and/or iii) in accordance with the terms applicable for US Federal Government officers or employees acting as part of their official duties; on a worldwide, perpetual, irrevocable, royalty-free basis to BMJ Publishing Group Ltd ("BMJ") its licensees and where the relevant Journal is co-owned by BMJ to the co-owners of the Journal, to publish the Work in this journal and any other BMJ products and to exploit all rights, as set out in our licence.

The Submitting Author accepts and understands that any supply made under these terms is made by BMJ to the Submitting Author unless you are acting as an employee on behalf of your employer or a postgraduate student of an affiliated institution which is paying any applicable article publishing charge ("APC") for Open Access articles. Where the Submitting Author wishes to make the Work available on an Open Access basis (and intends to pay the relevant APC), the terms of reuse of such Open Access shall be governed by a Creative Commons licence – details of these licences and which Creative Commons licence will apply to this Work are set out in our licence referred to above.

Other than as permitted in any relevant BMJ Author's Self Archiving Policies, I confirm this Work has not been accepted for publication elsewhere, is not being considered for publication elsewhere and does not duplicate material already published. I confirm all authors consent to publication of this Work and authorise the granting of this licence.

For peer review only

The association between relative income price and smoking initiation among adolescents in Ghana

Micheal Kofi Boachie[1, 2, 3], Mustapha Immurana[4], Ernest N. Tingum[2, 5], Noreen Dadirai Mdege[6], Hana Ross[2]**

[1] Department of Health Policy Planning and Management, School of Public Health, University of Health and Allied Sciences, Ho, Ghana; mkboachie@gmail.com

[2] Research Unit on the Economics of Excisable Products, School of Economics, University of Cape Town, Cape Town, South Africa; hana.ross@uct.ac.za

[3] SAMRC Centre for Health Economics and Decision Science, School of Public Health, University of the Witwatersrand, Johannesburg 2193, South Africa.

[4] Institute of Health Research, University of Health and Allied Sciences, Ho, Ghana; mustaphaimmurana@gmail.com

[5] Department of Economics, University of Namibia, Windhoek, Namibia; ngehernest@gmail.com

[6] Department of Health Sciences, University of York, Heslington, United Kingdom; noreen.mdege@york.ac.uk

** Correspondence: mkboachie@gmail.com; Tel.: +233 50 258 9141.

Word count:

Abstract: 236

Introduction: 678

Materials and Methods: 1191

Results: 648

Discussion and Conclusion: 464

Total word count: 2952

Number of References: 47

For peer review only - http://bmjopen.bmj.com/site/about/guidelines.xhtml

For peer review only

Abstract:

Objective: Many smokers initiate smoking during adolescence. Making tobacco products less affordable is one of the best ways to control tobacco use. Studies on the effect of relative income price (RIP (i.e., affordability)) of cigarettes on smoking initiation are scarce in low- and middle-income countries, especially in Sub-Saharan Africa where data are limited. The goal of this study is to examine the effect of cigarette RIP on adolescent smoking initiation in Ghana.

Setting: The study uses a pseudo-longitudinal dataset constructed from the Global Youth Tobacco Surveys (GYTS) and RIP for the most sold cigarette brand in Ghana.

Participants: The GYTS is a national survey on adolescents.

Primary and secondary outcome: Effect of RIP on adolescent smoking initiation in Ghana.

Results: We find that the probability of smoking initiation falls significantly in response to a higher RIP, with an elasticity of -0.374 (95% CI: -0.692 to -0.057) for the overall or unmatched sample and -0.547 (95% CI: -0.868 to -0.226) for the matched sample. The RIP elasticity for females (-0.685) (95% CI: -1.221 to -0.149) is statistically significant at 5% in the unmatched sample. In the matched sample, the elasticity is statistically significant at 1% for both males (-0.544) (95% CI: -0.864 to -0.224) and females (-0.551) (95% CI: -0.874 to -0.229).

Conclusion: The affordability (RIP) of cigarettes is negatively related to the probability of smoking initiation among adolescents. Raising tobacco taxes in line with income growth would make cigarettes less affordable and dissuade adolescents from initiating smoking.

Keywords: prices; taxes; smoking initiation; adolescents; relative income price

For peer review only - http://bmjopen.bmj.com/site/about/guidelines.xhtml

Strengths and Limitations of the study

- Research shows that higher cigarette prices reduce smoking and that making cigarettes less affordable is one of the best ways to control tobacco use.
- The impact of cigarette prices on smoking initiation is still being debated. Only few studies investigated the impact of cigarette relative income price (RIP), a proxy for their affordability, on smoking initiation. There is no such study using data from Sub-Saharan Africa.
- We examined the link between cigarette RIP and adolescent smoking initiation in Ghana and found an inverse relationship. This means that cigarette prices need to grow faster than inflation and income in order for them to have a negative impact on smoking uptake.
- The findings may not be generalized to other countries since it uses a single country data.

For peer review only

1. INTRODUCTION

Tobacco use is one of the major risk factors for many non-communicable diseases such as lung cancer and ischemic heart disease,[1 2] and it accounts for over eight million deaths annually worldwide.[3] Tobacco use also imposes huge financial burdens on households and governments.[4 5] The majority (80-90%) of the one billion adult smokers globally began the habit during their adolescence.[6] The current smoking prevalence in Ghana is still low relative to other African countries (3.2% among adults aged 15 years and older,[7] and 6.4% among students aged 13-15 years [8]). However, the number of smokers is predicted to increase from 1.3 million to 1.7 million (i.e., by about 30%) between 2020 and 2025.[9] The expected increase in the number of smokers will be partly driven by initiation among adolescents, therefore lowering initiation is key to slowing down the tobacco epidemic in Ghana.

Empirical evidence shows that tobacco consumption (initiation and intensity) is significantly inversely related to price.[10-12] In addition to being the most cost-effective measure to reduce tobacco use, increasing the taxes of tobacco products generates revenue for governments.[13-15] Nevertheless, economic factors such as income growth can negatively affect the response of tobacco consumption to tax/price.[16 17] Increasing tobacco prices can be more effective in reducing tobacco consumption if it reduces affordability.[18] Affordability (RIP) elasticity which measures the sensitivity of consumers to real changes in both price and income, can therefore be a useful parameter to explain and predict the sensitivity of consumers to tobacco tax and price policies even in the presence of income growth.[17] This is particularly important for tobacco control measures aimed at adolescents because they are more price sensitive than adults,[12 19] for instance in Ghana where 71.3% of current cigarette smokers aged 13-15 buy their own cigarettes.[8]

For peer review only - http://bmjopen.bmj.com/site/about/guidelines.xhtml

Some Sub-Saharan African (SSA) studies, albeit few, have demonstrated that increasing higher cigarette prices reduces smoking prevalence and intensity of use.[12 20-25] However, there is a scarcity of studies on the relationship between cigarette prices and smoking initiation in the SSA context. One study, using data from 48 countries, including four from SSA (Kenya, Nigeria, Senegal, and South Africa), concluded that higher cigarette prices reduce smoking initiation in early youth, with girls being more responsive than boys.[26] However, findings from the SSA countries were not reported separately from the overall study findings. A study in South Africa reported a significant reduction in regular smoking initiation among males due to higher cigarette prices, but not among females [10]. Another study in Nigeria and Ghana concluded that increasing cigarette prices resulted in a reduction in both 30-day cigarette smoking and cigarette smoking onset in both countries.[27]

Ghana has implemented a number of tobacco tax changes over the last 20 years. For example, it introduced a specific excise tax in 2007 and subsequently switched to an ad valorem tax structure in 2010. These changes have implications for the retail prices of tobacco products (e.g., cigarettes), and the affordability of smoking. However, to our knowledge, no study has analyzed the impact of cigarette affordability on smoking initiation in Ghana.

We address this critical evidence gap by examining the association between cigarette affordability and smoking initiation among adolescents in Ghana. Our analysis addresses the potential endogeneity of price as a driver of cigarette demand, as well as the fact that some people will never choose to smoke or use any form of tobacco, for example, for reasons of health or religious belief. We also examine potential sex differences in the association between affordability and cigarette smoking initiation. An understanding of these dynamics is key to the implementation of context-specific tobacco and non-communicable disease control policies in Ghana. We hypothesize that affordability of cigarettes in Ghana is related to smoking initiation among youth Ghana while taking into account sex, age, parents' and

For peer review only - http://bmjopen.bmj.com/site/about/guidelines.xhtml

friends' smoking status, being offered a cigarette for free, family/class discussion about tobacco, exposure to antismoking messages, and exposure to tobacco advertisements. These controls are based on variables that are known, empirically or theoretically, to be associated with smoking initiation, including in low- and middle-income countries.[27 28]

2. MATERIALS AND METHODS

2.1. Data and variables

We make use of three waves (2000, 2006 and 2009) of the Global Youth Tobacco Survey (GYTS) and RIP (affordability) data (1991–2009) to analyze the effect of affordability on smoking initiation among adolescents in Ghana. The World Health Organization defines adolescents as young people between the ages of 10 and 19 years. The GYTS questionnaire specifies ages from 11 years or younger to 17 years or higher. For the purposes of this study, we classify respondents as adolescents, youth or young people. The terms are used interchangeably in the study. The GYTS is a school-based survey developed to enhance the capacity of countries to monitor tobacco use among the youth, as well as implement and evaluate tobacco control and prevention programs.[29] These data provide representative trends of tobacco use among adolescents. The GYTS is a cross-sectional survey and does not follow individuals over time, but provides data on smoking patterns among adolescents. In countries where it is conducted at regular intervals, it allows the monitoring of trends over time. We are aware of the 2017 GYTS for Ghana, but we do not include it in the analysis due to inconsistencies in the questions asked, compared to those in previous GYTS surveys.

In Ghana, the GYTS sample is drawn using a two-stage cluster-sampling design.[29-31] Schools are selected with probability proportional to school enrolment size during the first stage, and then classes within participating schools are selected as a systematic equal probability sample

For peer review only - http://bmjopen.bmj.com/site/about/guidelines.xhtml

with a random start during the second stage. All students in the selected classes are eligible to participate in the survey.

The Ghana GYTS questionnaire captures information on the use of tobacco products such as cigarettes and shisha. The data also include parental and peer smoking habits, perception about tobacco use (e.g., weight gain, health effects, and ease of quitting), amount spent on tobacco in the last 30 days before the survey, and second-hand smoking (SHS).[32] Studies vary widely on the way they define or measure smoking initiation.[33-35] For the GYTS, smoking initiation is measured using the definition of a lifetime smoker, i.e., a person who has ever tried smoking, *even one or two puffs of a cigarette*.[27 35 36] Thus, for our study, smoking initiation (dependent variable) is a dichotomous variable generated from the following GYTS question where students answer Yes/No: *Have you ever tried or experimented with cigarette smoking, even one or two puffs?*.[27]

The main independent variable is the affordability index or the RIP, measured as the percentage of GDP per capita (per capita income) required to buy 100 packs of 2000 cigarettes.[17 18 37 38] Affordability is a relative measure and is calculated using nominal prices and nominal GDP per capita, or real prices and real GDP per capita. Data on per capita income are obtained from the World Bank's World Development Indicators,[39] and those of average cigarette prices (for the most-sold brand) come from the WHO [40] and relevant publications of the tobacco industry and the Government of Ghana. We calculate RIP following methods used by preceding studies, with a lower affordability index (RIP) indicating that cigarettes have become more affordable and a higher value indicating that cigarettes have become less affordable relative to the reference year.[17 18 37 38] The WHO uses the same approach to obtain its affordability index. However, the data from WHO cover only the period 2008 – 2018. Other independent variables are sex, age, parents' and friends' smoking status, whether offered cigarettes for free, family/class discussion about tobacco,

For peer review only - http://bmjopen.bmj.com/site/about/guidelines.xhtml

exposure to antismoking messages, and exposure to tobacco advertisements. These variables are selected as they have been shown, theoretically or empirically, to be associated with smoking initiation.[27 28] Except age and RIP, which are continuously measured, all variables are dichotomous.

2.2. Data analysis

We construct a pseudo-longitudinal dataset based on current age and age at first puff. In doing this, we create a historical dataset starting from age 8 (age-at-risk criteria) and following the person until s/he initiates smoking. We then link the RIP (affordability index) to this pseudo-longitudinal dataset. Although the GYTS data contain adolescents whose first puff was at age 7 or younger, we assume that a student is at risk at age 8 and exits the sample once smoking is initiated.[10 27 41 42] We choose age 8 as the age-at-risk because that is the age at which the child is relatively developed and able to start out-of-home interaction with peers, according to previous studies.[42] Students who started smoking before reaching age 8 were therefore excluded from the pooled sample and not followed.

Our statistical technique is duration or event history analysis where the timing of transition into initiation is a function of the probability of initiating in period t conditional on not having experienced a transition until period t, known as the hazard rate.[10] Following Vellios and Van Walbeek [10] and Guindon, et al. [42], we employ the discrete time-hazard model, with logit specification (see equation 1), to study the association between RIP (affordability) and smoking initiation among adolescents.

$$\Pr(Initiation = 1 \mid X') = \beta_0 + \beta_1 RIP + \beta_i X' \quad \ldots (1),$$

where Initiation is defined as first cigarette puff, RIP is the affordability index, X' is a vector of other independent variables affecting smoking initiation among adolescents, and β is a vector of the regression coefficients. The predictors, X' , represent age, sex, whether offered

For peer review only - http://bmjopen.bmj.com/site/about/guidelines.xhtml

free cigarette, parental and peer smoking status, family/class discussion on the dangers of tobacco, exposure to tobacco advertisements, and hearing of antismoking messages. We report odds ratios (ORs), and the statistical level of significance is set at $p \leq 0.05$. OR <1 implies that when a higher share of income is required to buy 2000 cigarettes (cigarettes are less affordable), the risk of smoking initiation declines, and vice versa. The partial derivative of equation 1 with respect to RIP gives the affordability elasticity.

To check the robustness of the logistic regression estimates, we employ a propensity score matching (PSM) technique to match ever-smokers to never-smokers based on the propensity scores. Our approach to matching follows previous studies.[41 43 44] The propensity scores are obtained by running a logit regression to estimate the probability of being a smoker based on the variables in equation 1, except RIP, and the predicted probabilities are then used to match ever-smokers to never-smokers. Using the neighborhood matching, ever-smokers are matched to their two nearest neighbors. After matching the sample, we re-estimate the logit model to assess the effect of affordability on the probability of initiating smoking, using GYTS weights on the matched sample.[43 44] With the matching approach, we are able to obtain the effect of affordability on the probability of initiating smoking among adolescent smokers and non-smokers who possess similar characteristics based on the propensity scores. This technique addresses issues of endogeneity and concerns relating to the fact that some never-smokers will never choose to smoke or use any form of tobacco irrespective of market conditions.[41] Further, we minimize the problem of endogeneity by not using self-reported prices.[42] Data analysis is conducted using STATA version 15.

Patient and Public Involvement:

No patient involved.

For peer review only - http://bmjopen.bmj.com/site/about/guidelines.xhtml

3. RESULTS

3.1. Descriptive statistics

A total of 20,202 adolescents were interviewed across the three GYTS waves. 54% of the respondents were male, while 76.47% were aged 15 years or less. 12.46% of the respondents in the three surveys had ever smoked (tabular results are available on request).

Given our age-at-risk criteria, 15,861 people were eligible for inclusion in our pseudo-longitudinal analysis. Of this number, 4.2% initiated smoking at some point between 1991 and 2009, and about 77% of smoking initiators did so before reaching age 16. Further, 67% of initiators were males. Overall, males represented 53.62% of the eligible respondents. Due to incomplete information on some of the variables, the number of people used in the regression varies. Characteristics of the sample are shown in Table 1.

Table 1: Descriptive statistics

Variable	Students, n = 15 861
Initiated smoking during the period	4.20%
RIP (Affordability)	19.87 (SD=6.53)
Offered free cigarettes	12.44%
Sex (male =1) sample	53.62%
At least one parent smoke	11.78%
Family/class discuss about tobacco	72.50%
At least a friend smoke	15.94%
Exposed to tobacco adverts	40.46%
Age (Years)	14.15 (SD=1.7)
Heard anti-smoking campaigns	74.64%
Age at initiation (Years)	11.95 (SD = 2.9)
Percentage of initiators before age 16	77%
Percentage of initiators who are males	67%

For peer review only - http://bmjopen.bmj.com/site/about/guidelines.xhtml

3.2. Regression Results

Results from the logit regressions for the unmatched and matched samples are reported in Table 2. The results show a statistically significant and negative relationship between RIP and smoking initiation. For instance, the results for the full unmatched sample (model 1, Table 2) show that OR on RIP is 0.98. This implies that a unit increase in affordability is associated with 0.98 odds of initiating smoking (OR = 0.98, p<0.05). Thus, an adolescent who is subjected to a unit increase in RIP has 2% (i.e., 1 – 0.98) × 100 = 2%)) lower probability of initiating smoking than his/her counterpart who is not subjected to the same increase. Note that these results are not elasticities. Females have a 39% (i.e., 1 – 0.613) × 100 = 39%)) lower probability of initiating smoking (OR = 0.613, p<0.01) compared to males in the unmatched sample.

Table 2: Effect of RIP on smoking initiation among adolescents

	**(Unmatched Sample)**	**Matched Sample**
	**Model 1**	**Model 2**
VARIABLES	Odds Ratio	Odds Ratio
RIP	0.980**	0.971***
	(0.008)	(0.009)
Sex (Female =1)	0.613***	0.744**
	(0.074)	(0.091)
Offered free cigarette	1.444**	0.717**
	(0.211)	(0.101)
At least one parent smokes	2.097***	0.872
	(0.276)	(0.107)
Family/class discussion on tobacco	1.001	1.422***
	(0.133)	(0.190)
At least one friend smokes	4.049***	1.082
	(0.513)	(0.126)
Exposed to tobacco adverts	1.136	1.086
	(0.139)	(0.132)
Age	0.393	1.075
	(0.197)	(0.609)
Antismoking message	1.352	1.486**
	(0.218)	(0.238)
Constant	0.885	0.113

For peer review only - http://bmjopen.bmj.com/site/about/guidelines.xhtml

	(3.169)	(0.460)
Observations (person-period)	106,673	7,469
Number of people	15,201	1,189
Pseudo R-squared	0.0822	0.0304
Chi-squared	437.3***	75.41***

Standard errors in parentheses

*** p<0.01, ** p<0.05, * p<0.1

Other significant factors that influence smoking initiation in our sample include whether the adolescent's parents (OR = 2.097, p<0.01) or friends (OR = 4.049, p<0.01) smoke. In addition, adolescents who are offered free cigarettes have a high probability of initiating smoking (OR = 1.444, p<0.05) compared to those who receive no such offer.

In the matched sample (model 2, Table 2), 611 ever-smokers were matched to their two nearest neighbors (578 never-smokers) which produced a sample of 1189 adolescents with similar characteristics. An adolescent subjected to a unit increase in RIP has a 0.97 times lower chance of initiating smoking (OR = 0.971, p<0.01) compared to those not exposed to the same increase in RIP. Getting free cigarettes (OR = 0.717, p<0.05), family/class discussion on tobacco (OR = 1.422, p<0.01), and hearing antismoking messages (OR = 1.486, p<0.05) are all found to be statistically significant in influencing smoking initiation in the matched sample. However, the odds for these variables are contrary to *a priori* expectations. Similarly, in the matched sample (Table 2), the likelihood of initiating smoking among females is lower. The results imply that females have about a 26% lower probability of initiating smoking (OR = 0.744, p<0.05) than males.

3.3. Affordability Elasticities

In the unmatched sample, the estimated affordability elasticity is -0.374 [CI: -0.692 to -0.057] and it is statistically significant at the 5% level. By sex, the affordability elasticity is -0.238

For peer review only - http://bmjopen.bmj.com/site/about/guidelines.xhtml

For peer review only

for males and -0.685 for females, but only that of females is statistically significant (at the 5% level). The results are presented in Table 3.

Table 3: Affordability Elasticity estimates (unmatched sample)

VARIABLE	Both sexes	Males	Female
RIP	-0.374**	-0.238	-0.685**
	(0.162)	(0.199)	(0.274)
95% CI	-0.692 to -0.057	-0.628 to 0.153	-1.221 to -0.149
Observations	106,673	55,396	51,277

Standard errors in parentheses. *** p<0.01, ** p<0.05, * p<0.1

Table 4 presents the affordability elasticity for the matched sample. The overall elasticity is -0.547 [CI: -0.868 to -0.226] for both sexes (Table 4), which is similar to that of the unmatched sample in Table 4. For males, a percentage increase in RIP reduces the likelihood of initiating smoking by 0.544% and is statistically significant at 1%. Among females, a percentage increase in RIP is associated with a 0.551% lower probability of smoking initiation.

Table 4: Affordability Elasticity estimates after matching.

Variables	Both sexes	Males	Females
RIP	-0.547***	-0.544***	-0.551***
	(0.163)	(0.163)	(0.164)
95% CI	-0.868 to -0.226	-0.864 to -0.224	-0.874 to -0.229
Observations	7469	4522	2947

Standard errors in parentheses. *** p<0.01, ** p<0.05, * p<0.1

The mean and median standardized difference for the covariates used in matching show that the matching satisfies the balancing test (results not reported). The mean and median standardized difference is 2.3%, which is below the normal 10% threshold. Therefore, the balancing property is satisfied.

For peer review only

5. DISCUSSION AND CONCLUSION

In this study, increasing the RIP of cigarettes is significantly associated with a lower probability of initiating smoking. This finding is consistent with international literature, including the few existing studies in SSA that have reported that making cigarettes less affordable lowers the likelihood of smoking initiation among young people.[17 18 26 27] In addition, the results from the unmatched sample suggest that males are not responsive to changes in RIP whilst females are. Nevertheless, in the matched sample analysis both boys and girls were responsive to changes in RIP.

Parental smoking increased the odds of smoking initiating. This points to the parental influence on the lifestyle of adolescents. Adolescents whose parents smoke may perceive smoking as acceptable behavior. Previous studies have reported similar findings.[10 27 28 45] The odds of smoking initiation higher for those whose friends smoke. This points to the influence of peers, and is consistent, for example, with Mak, et al. [28]. Those who were offered free cigarettes by sales representatives were more likely to initiate smoking. All forms of tobacco promotion and advertising were banned in Ghana in 1982. However, the tobacco industry seems to be breaking these laws, because 12.44% of youth reported being offered a cigarette for free. This observation, together with our results, suggest the need to strengthen the enforcement of the existing ban on all forms of tobacco advertising and promotion in Ghana.

This study has several limitations. The GYTS is a self-reporting survey, which means the responses are prone to recall bias even in cases where the adolescents are required to answer questions about events that occurred in the past 30 days. For instance, students may not recall the exact age at which they tried their first puff. There is also a social desirability bias when self-reporting behaviors such as smoking, especially among females. The weakness of our measure of smoking initiation is that it may not predict regular smoking behavior well.[34 35] In

For peer review only - http://bmjopen.bmj.com/site/about/guidelines.xhtml

addition, there are other important factors affecting smoking uptake among the youth that are not included in this study. For instance, changing community norms regarding smoking, the enforcement of laws regarding the sale of cigarettes to minors, and changes in the social image of smoking are key factors that may influence smoking participation,[46 47] but are not included in the models estimated.

In conclusion, making cigarettes less affordable is associated with a lower probability of smoking initiation among adolescents in Ghana. This supports the use of price measures, through higher excise taxes, as effective strategies to decrease smoking initiation among adolescents in Ghana. With rising incomes, tobacco taxes need to be adjusted regularly to ensure that cigarettes become less affordable over time in order to discourage young people from initiating smoking and to encourage smokers to quit.

Author Contributions: Conceptualization, M.K.B.; methodology, M.K.B; software, M.K.B.; validation, M.K.B.., M.I., E.N.T., N.D.M., and H.R.; formal analysis, M.K.B.; investigation, M.K.B., M.I., E.N.T., N.D.M., and H.R.; data curation, M.K.B; writing—original draft preparation, M.K.B., M.I., E.N.T., N.D.M., and H.R.; writing—review and editing, M.K.B.., M.I., E.N.T., N.D.M., H.R.; project administration, M.K.B.; supervision, H.R.; funding acquisition, H.R. All authors have read and approved the manuscript.

Funding: Funding for this study was provided by Cancer Research UK (Grant number: C62640/A24723) and International Development Research Centre (Grant Number: 108820-001). Funding for ENT and NDM is acknowledged from the Tobacco Control Capacity Programme (Grant Number: MR/P027946/2) supported by UK Research and Innovation (UKRI) with funding from the Global Challenges Research Fund (GCRF). The funders had no role in study design, data collection and analysis, or preparation of the manuscript.

Conflicts of Interest: The authors declare that they have no conflict of interest.

Research Ethics Approval: Human Participants

For peer review only - http://bmjopen.bmj.com/site/about/guidelines.xhtml

This study does not involve human participants.

Research Ethics Approval: Animals

This study does not involve animal subjects.

Patient consent for publication: No patient involved.

Data Availability statement: The publicly available data can be accessed: https://nccd.cdc.gov/GTSSDataSurveyResources/Ancillary/DataReports.aspx?CAID=2. The relative income price data are available from the authors on reasonable request.

REFERENCES

1. Doll R, Peto R, Boreham J, et al. Mortality in relation to smoking: 50 years' observations on male British doctors. *Bmj* 2004;328(7455):1519.
2. Winkler V, Mangolo NJ, Becher H. Lung cancer in South Africa: a forecast to 2025 based on smoking prevalence data. *BMJ open* 2015;5(3):e006993.
3. World Health Organization. Tobacco: Key Facts. 2020 [updated 27 May 2020. Available from: https://www.who.int/news-room/fact-sheets/detail/tobacco accessed 22 November 2020.
4. Goodchild M, Nargis N, d'Espaignet ET. Global economic cost of smoking-attributable diseases. *Tobacco Control* 2018;27(1):58-64.
5. Boachie MK, Rossouw L, Ross H. The Economic Cost of Smoking in South Africa, 2016. *Nicotine Tob Res* 2021;23(2):286 - 93. doi: 10.1093/ntr/ntaa162
6. USDHHS. Preventing tobacco use among youth and young adults: A report of the surgeon general 2012 [Available from: http://www.surgeongeneral.gov/library/reports/preventing-youth-tobacco-use/full-report.pdf (accessed: Jan 3, 2019).

For peer review only - http://bmjopen.bmj.com/site/about/guidelines.xhtml

7. World Health Organization. WHO global report on trends in prevalence of tobacco use 2000-2025. 3rd ed. Geneva: World Health Organization, 2019.

8. CDC, WHO, MOH. GLOBAL YOUTH TOBACCO SURVEY: Factsheet, Ghana 2017, 2018.

9. World Health Organization. WHO global report on trends in prevalence of tobacco smoking 2015. Geneva: World Health Organization, 2015.

10. Vellios N, Van Walbeek C. Determinants of regular smoking onset in South Africa using duration analysis. *BMJ Open* 2016;6(7):e011076.

11. IARC. Effectiveness of tax and price policies for tobacco control. In: Cancer. IAfRo, ed. USA: International Agency for Research on Cancer, 2011.

12. Boachie MK, Ross H. Determinants of smoking intensity in South Africa: evidence from township communities. *Preventive Medicine Reports* 2020:101099.

13. Van Walbeek C. A simulation model to predict the fiscal and public health impact of a change in cigarette excise taxes. *Tobacco control* 2010;19(1):31-36.

14. Yurekli A, de Beye J. Design and administer tobacco taxes. *World Bank economics of tobacco toolkit* 2001(4)

15. Van Walbeek C. Raising Additional Government Revenues in Ghana by Raising the Excise Tax on Tobacco and Alcohol Washington, D.C.: WBG Global Tobacco Control Program, World Bank Group; 2014 [Available from: http://documents.worldbank.org/curated/en/133561561707742558/pdf/Raising-Additional-Government-Revenues-in-Ghana-by-Raising-the-Excise-Tax-on-Tobacco-and-Alcohol.pdf accessed 15 July 2019.

16. He Y, Shang C, Chaloupka FJ. The association between cigarette affordability and consumption: An update. *PLOS ONE* 2018;13(12):e0200665. doi: 10.1371/journal.pone.0200665

17. Nargis N, Stoklosa M, Shang C, et al. Price, Income, and Affordability as the Determinants of Tobacco Consumption: A Practitioner's Guide to Tobacco Taxation. *Nicotine and Tobacco Research* 2021;23(1):40-47.

For peer review only - http://bmjopen.bmj.com/site/about/guidelines.xhtml

18. Blecher E, Van Walbeek C. An international analysis of cigarette affordability. *Tobacco Control* 2004;13(4):339-46.

19. Ding A. Youth are more sensitive to price changes in cigarettes than adults. *The Yale journal of biology and medicine* 2003;76(3):115.

20. Ho L-M, Schafferer C, Lee J-M, et al. The effect of cigarette price increases on cigarette consumption, tax revenue, and smoking-related death in Africa from 1999 to 2013. *International journal of public health* 2017;62(8):899-909.

21. Chelwa G, van Walbeek C. Does cigarette demand respond to price increases in Uganda? Price elasticity estimates using the Uganda National Panel Survey and Deaton's method. *BMJ open* 2019;9(3):e026150. doi: 10.1136/bmjopen-2018-026150 [published Online First: 2019/03/23]

22. Stoklosa M, Goma F, Nargis N, et al. Price, tax and tobacco product substitution in Zambia: findings from the ITC Zambia Surveys. *Tob Control* 2019;28(Suppl 1):s45-s52. doi: 10.1136/tobaccocontrol-2017-054037 [published Online First: 2018/03/27]

23. Mukong AK, Tingum EN. The demand for cigarettes: New evidence from South Africa. *Development Southern Africa* 2020;37(1):40-54.

24. Tingum EN, Mukong AK, Mdege N. The effects of price and non-price policies on cigarette consumption in South Africa. *Tob Induc Dis* 2020;18(July) doi: 10.18332/tid/123424

25. Kidane A, Mduma J, Naho A, et al. The Demand for Cigarettes in Tanzania and Implications for Tobacco Taxation Policy. *Advances in economics and business* 2015;3(10):428.

26. Kostova D. A (nearly) global look at the dynamics of youth smoking initiation and cessation: the role of cigarette prices. *Applied Economics* 2013;45(28):3943-51.

27. Asare S, Stoklosa M, Drope J, et al. Effects of Prices on Youth Cigarette Smoking and Tobacco Use Initiation in Ghana and Nigeria. *International Journal of Environmental Research and Public Health* 2019;16(17):3114.

28. Mak K-K, Ho S-Y, Day JR. Smoking of parents and best friend—independent and combined effects on adolescent smoking and intention to initiate and quit smoking. *Nicotine Tob Res* 2012;14(9):1057-64.

For peer review only

For peer review only - http://bmjopen.bmj.com/site/about/guidelines.xhtml

29. Warren CW. Tobacco use among youth: a cross country comparison. *Tobacco Control* 2002;11(3):252-70.

30. Wellington E. Ghana Global Youth Tobacco Survey (GYTS), Factsheet. 2006 [Available from: http://www.afro.who.int/en/downloads/docdownload/1887-ghana-gyts-2006-factsheet.html (Retrieved Jan 28, 2019).

31. Cadmus EO, Ayo-Yusuf OA. The effect of smokeless tobacco use and exposure to cigarette promotions on smoking intention among youths in Ghana. *Cogent Medicine* 2018;5(1):1531459.

32. CDC and WHO. Ghana global youth tobacco survey. Atlanta, United States: Centers for Disease Control and Prevention (CDC). 2006.

33. Freedman KS, Nelson NM, Feldman LL. Smoking initiation among young adults in the United States and Canada, 1998-2010: a systematic review. *Preventing chronic disease* 2012;9

34. Azagba S, Baskerville NB, Minaker L. A comparison of adolescent smoking initiation measures on predicting future smoking behavior. *Preventive medicine reports* 2015;2:174-77.

35. Reidpath DD, Davey TM, Kadirvelu A, et al. Does one cigarette make an adolescent smoker, and is it influenced by age and age of smoking initiation? Evidence of association from the US Youth Risk Behavior Surveillance System (2011). *Preventive medicine* 2014;59:37-41.

36. Centers for Disease Control Prevention. Selected cigarette smoking initiation and quitting behaviors among high school students--United States, 1997. *MMWR Morbidity and mortality weekly report* 1998;47(19):386.

37. Blecher E. Targeting the affordability of cigarettes: a new benchmark for taxation policy in low-income and-middle-income countries. *Tobacco control* 2010;19(4):325-30.

38. Nargis N, Stoklosa M, Drope J, et al. Trend in the affordability of tobacco products in Bangladesh: findings from the ITC Bangladesh Surveys. *Tobacco control* 2019;28(Suppl 1):s20-s30.

For peer review only - http://bmjopen.bmj.com/site/about/guidelines.xhtml

39. World Bank. World Development Indicators.: World Bank; 2019 [Available from: https://databank.worldbank.org/source/world-development-indicators. accessed 01 Jan 2020.

40. World Health Organization. WHO report on the global tobacco epidemic 2019: Offer help to quit tobacco use. 2019

41. Dauchy E, Ross H. The Effect of Price and Tax Policies on the Decision to Smoke among Men in Kenya. *Addiction* 2019;114(7):1249-63.

42. Guindon GE, Paraje GR, Chávez R. Prices, inflation, and smoking onset: the case of Argentina. *Economic Inquiry* 2018;56(1):424-45.

43. Austin PC, Jembere N, Chiu M. Propensity score matching and complex surveys. *Statistical methods in medical research* 2018;27(4):1240-57.

44. DuGoff EH, Schuler M, Stuart EA. Generalizing observational study results: applying propensity score methods to complex surveys. *Health services research* 2014;49(1):284-303.

45. Gilman SE, Rende R, Boergers J, et al. Parental smoking and adolescent smoking initiation: an intergenerational perspective on tobacco control. *Pediatrics* 2009;123(2):e274-e81.

46. Gilpin EA, Pierce JP. Trends in adolescent smoking initiation in the United States: is tobacco marketing an influence? *Tobacco Control* 1997;6(2):122-27.

47. Cawley J, Markowitz S, Tauras J. Obesity, cigarette prices, youth access laws and adolescent smoking initiation. *Eastern Economic Journal* 2006;32(1):149-70.

For peer review only

For peer review only - http://bmjopen.bmj.com/site/about/guidelines.xhtml

BMJ Open

Effect of relative income price on smoking initiation among adolescents in Ghana: evidence from pseudo-longitudinal data

Journal:	*BMJ Open*
Manuscript ID	bmjopen-2021-054367.R1
Article Type:	Original research
Date Submitted by the Author:	11-Dec-2021
Complete List of Authors:	Boachie, Micheal Kofi; University of Health and Allied Sciences, Health Policy Management<br>Immurana, Mustapha; University of Health and Allied Sciences, Institute of Health Research<br>Tingum, Ernest; University of Cape Town, Economics<br>Mdege, Noreen; University of York, Health Sciences<br>Ross, Hana; University of Cape Town, School of Economics
**Primary Subject Heading**:	Global health
Secondary Subject Heading:	Health economics, Global health, Public health, Smoking and tobacco
Keywords:	HEALTH ECONOMICS, PUBLIC HEALTH, SOCIAL MEDICINE

SCHOLARONE™
Manuscripts

I, the Submitting Author has the right to grant and does grant on behalf of all authors of the Work (as defined in the below author licence), an exclusive licence and/or a non-exclusive licence for contributions from authors who are: i) UK Crown employees; ii) where BMJ has agreed a CC-BY licence shall apply, and/or iii) in accordance with the terms applicable for US Federal Government officers or employees acting as part of their official duties; on a worldwide, perpetual, irrevocable, royalty-free basis to BMJ Publishing Group Ltd ("BMJ") its licensees and where the relevant Journal is co-owned by BMJ to the co-owners of the Journal, to publish the Work in this journal and any other BMJ products and to exploit all rights, as set out in our licence.

The Submitting Author accepts and understands that any supply made under these terms is made by BMJ to the Submitting Author unless you are acting as an employee on behalf of your employer or a postgraduate student of an affiliated institution which is paying any applicable article publishing charge ("APC") for Open Access articles. Where the Submitting Author wishes to make the Work available on an Open Access basis (and intends to pay the relevant APC), the terms of reuse of such Open Access shall be governed by a Creative Commons licence – details of these licences and which Creative Commons licence will apply to this Work are set out in our licence referred to above.

Other than as permitted in any relevant BMJ Author's Self Archiving Policies, I confirm this Work has not been accepted for publication elsewhere, is not being considered for publication elsewhere and does not duplicate material already published. I confirm all authors consent to publication of this Work and authorise the granting of this licence.

For peer review only

Effect of relative income price on smoking initiation among adolescents in Ghana: evidence from pseudo-longitudinal data

Micheal Kofi Boachie[1, 2], Mustapha Immurana[3], Ernest N. Tingum[2, 4], Noreen Dadirai Mdege[5], Hana Ross[2]**

[1] SAMRC/Wits Centre for Health Economics and Decision Science – PRICELESS SA, School of Public Health, University of the Witwatersrand, Johannesburg 2193, South Africa; mkboachie@gmail.com

[2] Research Unit on the Economics of Excisable Products, School of Economics, University of Cape Town, Cape Town, South Africa; hana.ross@uct.ac.za

[3] Institute of Health Research, University of Health and Allied Sciences, Ho, Ghana; mimmurna@uhas.edu.gh; mustaphaimmurana@gmail.com

[4] Department of Economics, University of Namibia, Windhoek, Namibia; ngehernest@gmail.com

[5] Department of Health Sciences, University of York, Heslington, United Kingdom; noreen.mdege@york.ac.uk

** Correspondence: mkboachie@gmail.com; Tel.: +27 845 644 650.

Word count:

Abstract: 290

Introduction: 741

Materials and Methods: 1517

Results: 1330

Discussion and Conclusion: 508

Total word count: 4386

Number of References: 54

For peer review only - http://bmjopen.bmj.com/site/about/guidelines.xhtml

Abstract:

Objective: Many smokers initiate smoking during adolescence. Making tobacco products less affordable is one of the best ways to control tobacco use. Studies on the effect of relative income price (RIP (i.e., affordability)) of cigarettes on smoking initiation are scarce in low- and middle-income countries, especially in Sub-Saharan Africa where data are limited. The goal of this study is to examine the effect of cigarette RIP on adolescent smoking initiation in Ghana.

Setting: The study uses a pseudo-longitudinal dataset constructed from the Global Youth Tobacco Surveys (GYTS (2000-2009 and 2017)) and RIP for the most sold cigarette brand in Ghana.

Participants: The GYTS is a national survey on adolescents.

Primary and secondary outcome: Effect of RIP on adolescent smoking initiation in Ghana.

Results:

Using the GYTS 2000-2009 data, we find that the probability of smoking initiation falls significantly in response to a higher RIP, with an elasticity of -0.372 (95% CI: -0.701 to -0.042) for the unmatched sample and -0.490 (95% CI: -0.818 to -0.161) for the matched sample. The RIP elasticity for females [(-0.888) (95% CI: -1.384 to -0.392) and (-0.928) (95% CI: -1.434 to -0.422)] is statistically significant at 1% in both the unmatched and the matched samples, respectively, while the RIP elasticity for males is statistically insignificant in the 2000 - 2009 surveys. Analysis of the 2017 GYTS shows a similar outcome: a negative relationship between RIP and smoking initiation, and the results are statistically significant for both male and female, and for both matched and unmatched samples.

For peer review only - http://bmjopen.bmj.com/site/about/guidelines.xhtml

For peer review only

Conclusion: The affordability (RIP) of cigarettes is negatively related to the probability of smoking initiation among adolescents in Ghana. Raising tobacco taxes in line with income growth would make cigarettes less affordable and dissuade adolescents from initiating smoking.

Keywords: prices; taxes; smoking initiation; adolescents; relative income price

For peer review only

Strengths and Limitations of the study

- This is the first study to explore the impact of Relative Income Price (i.e., affordability index) of cigarettes on youth smoking initiation in Sub-Saharan Africa.
- Our analysis controls for variables that are known, empirically or theoretically, to be associated with smoking initiation, and the relationship is tested using a pseudo-longitudinal dataset of 17 years.
- We also examine potential sex differences in the effect of affordability on cigarette smoking initiation: this is key to the implementation of tobacco control policies that confer adequate protection across both genders.
- Since GYTS data are available in many low- and middle-income countries (LMIC), our study provide a template on how to do analyses elsewhere in order to enhance our understating of the impact of cigarette affordability on smoking uptake in LMIC.

For peer review only - http://bmjopen.bmj.com/site/about/guidelines.xhtml

- The results are subject to self-reporting and recall biases as well as omitted variable bias due to lack of data on other factors affecting smoking uptake.

For peer review only

For peer review only - http://bmjopen.bmj.com/site/about/guidelines.xhtml

1. INTRODUCTION

Tobacco use is one of the major risk factors for many non-communicable diseases such as lung cancer and ischemic heart disease,[1 2] and it accounts for over eight million deaths annually worldwide.[3] Tobacco use also imposes huge financial burdens on households and governments.[4 5] The majority (80-90%) of the one billion adult smokers globally began the habit during their adolescence.[6] The current smoking prevalence in Ghana is still low relative to other African countries (3.2% among adults aged 15 years and older,[7] and 6.4% among students aged 13-15 years [8]). However, the number of smokers is predicted to increase from 1.3 million to 1.7 million (i.e., by about 30%) between 2020 and 2025.[9] The expected increase in the number of smokers will be partly driven by initiation among adolescents, therefore lowering initiation is key to slowing down the tobacco epidemic in Ghana.

Empirical evidence shows that tobacco consumption (initiation and intensity) is significantly inversely related to price.[10-12] In addition to being the most cost-effective measure to reduce tobacco use, increasing the taxes of tobacco products generates revenue for governments.[13-15] Nevertheless, economic factors such as income growth can negatively affect the response of tobacco consumption to tax/price.[16 17] Increasing tobacco prices can be more effective in reducing tobacco consumption if it reduces affordability.[18] Affordability (relative income price (RIP)) elasticity which measures the sensitivity of consumers to real changes in both price and income, can therefore be a useful parameter to explain and predict the sensitivity of consumers to tobacco tax and price policies even in the presence of income growth.[17] This is particularly important for tobacco control measures aimed at adolescents because they are more price sensitive than adults,[12 19] for instance in Ghana where 71.3% of current cigarette smokers aged 13-15 buy their own cigarettes.[8]

For peer review only - http://bmjopen.bmj.com/site/about/guidelines.xhtml

Some Sub-Saharan African (SSA) studies, albeit few, have demonstrated that increasing higher cigarette prices reduces smoking prevalence and intensity of use.[12 20-25] However, there is a scarcity of studies on the relationship between cigarette prices and smoking initiation in the SSA context. One study, using data from 48 countries, including four from SSA (Kenya, Nigeria, Senegal, and South Africa), concluded that higher cigarette prices reduce smoking initiation in early youth, with girls being more responsive than boys.[26] However, findings from the SSA countries were not reported separately from the overall study findings. A study in South Africa reported a significant reduction in regular smoking initiation among males due to higher cigarette prices, but not among females [10]. Another study in Nigeria and Ghana concluded that increasing cigarette prices resulted in a reduction in both 30-day cigarette smoking and cigarette smoking onset in both countries.[27]

Ghana has implemented a number of tobacco tax changes over the last 20 years. For example, it introduced a specific excise tax in 2007 and subsequently switched to an ad valorem tax structure in 2010.[28 29] At the same time, per capita income in Ghana has been growing at an average rate of 4.4% annually in the last decade.[30] These changes have implications for the retail prices of tobacco products (e.g., cigarettes), and the affordability of cigarettes or other tobacco products. However, to our knowledge, no study has analyzed the impact of cigarette affordability on smoking initiation in Ghana.

We address this critical evidence gap by examining the effect of cigarette affordability on smoking initiation among adolescents in Ghana using. We hypothesize that making cigarettes less affordable reduces the likelihood of smoking initiation among young people, and make use of the Global Youth Tobacco Survey (GYTS) data and other datasets to test that hypothesis. The control variables used are sex, age, parents', and friends' smoking status, being offered a cigarette for free, family/class discussion about tobacco, exposure to antismoking messages, and exposure to tobacco advertisements. These controls are based on

For peer review only - http://bmjopen.bmj.com/site/about/guidelines.xhtml

variables that are known, empirically or theoretically, to be associated with smoking initiation.[27 31]

Our analysis addresses the potential endogeneity of price, if any, as a driver of cigarette demand through i) using aggregate level prices and not self-reported prices[32], ii) the use of propensity score matching techniques and iii) the fact that some people will never choose to smoke or use any form of tobacco, for example, for reasons of health or religious belief[33 34] We also examine potential sex differences in the effect of affordability on cigarette smoking initiation. An understanding of these dynamics is key to the implementation of context-specific tobacco and non-communicable disease control policies in Ghana.

2. MATERIALS AND METHODS

2.1. Data and variables

We make use of three waves (2000, 2006 and 2009) of the GYTS and RIP (affordability) data (1991–2009) to analyze the effect of affordability on smoking initiation among adolescents in Ghana. The World Health Organization (WHO) defines adolescents as young people between the ages of 10 and 19 years. The GYTS questionnaire specifies ages from 11 years or younger to 17 years or higher for current age (i.e., age at the time of survey). It also captures age at first puff, which ranges from 7 years or younger to 16 years or older. For the purposes of this study, we classify respondents as adolescents, youth or young people. The terms are used interchangeably in the study. The GYTS is a school-based survey developed to enhance the capacity of countries to monitor tobacco use among the youth, as well as implement and evaluate tobacco control and prevention programs.[35] These data provide representative trends of tobacco use among adolescents. The GYTS is a cross-sectional survey and does not follow individuals over time, but provides data on smoking patterns among adolescents. In countries where it is conducted at regular intervals, it allows the monitoring of trends over time. We are

For peer review only - http://bmjopen.bmj.com/site/about/guidelines.xhtml

aware of the 2017 GYTS for Ghana, but we do not include it in the analysis of the pooled 2000 – 2009 surveys due to inconsistencies in the questions asked, compared to those in previous GYTS surveys. There is no other survey on adolescents in Ghana with comparable measures except the ones outlined. We analyze the 2017 GYTS separately while linking it with RIP data for 2008 – 2017 based on age-at-risk criteria (as done for the 2000 – 2009 surveys).

In Ghana, there was no law prohibiting the sale of cigarettes to minors until 2012 when restrictions on sale to persons below age 18 years were introduced.[36] The GYTS sample is drawn using a two-stage cluster-sampling design.[35 37 38] Schools are selected with probability proportional to school enrolment size during the first stage, and then classes within participating schools are selected as a systematic equal probability sample with a random start during the second stage. All students in the selected classes are eligible to participate in the survey.

The Ghana GYTS questionnaire captures information on the use of tobacco products such as cigarettes and shisha. The data also include parental and peer smoking habits, perception about tobacco use (e.g., weight gain, health effects, and ease of quitting), amount spent on tobacco in the last 30 days before the survey, and second-hand smoking (SHS).[39] Studies vary widely on the way they define or measure smoking initiation.[40-42] For the GYTS, smoking initiation is measured using the definition of a lifetime smoker, i.e., a person who has ever tried smoking, *even one or two puffs of a cigarette*.[27 42 43] Thus, for our study, smoking initiation (dependent variable) is a dichotomous variable generated from the following GYTS question where students answer Yes/No: *Have you ever tried or experimented with cigarette smoking, even one or two puffs?*.[27]

For peer review only - http://bmjopen.bmj.com/site/about/guidelines.xhtml

The main independent variable is the affordability index or the RIP, measured as the percentage of GDP per capita (per capita income) required to buy 100 packs of cigarettes (20 sticks per pack, in total 2,000 sticks).[17 18 44 45] Affordability is a relative measure and is calculated using nominal prices and nominal GDP per capita, or real prices and real GDP per capita. Data on per capita income are obtained from the World Bank's World Development Indicators,[30] and those of average cigarette prices (for the most-sold brand) come from the WHO, relevant publications of the tobacco industry[46] and the Government of Ghana[47]. Years with missing data on prices were interpolated using the formula:

$$P_{t-1} = \frac{P_t}{(1 + Tob.Inflation_t)} \quad \ldots\ldots\ldots\ldots\ldots\ldots (1)$$

where P_{t-1} is the previous year's price of cigarette, P_t is current price of cigarette and $Tob.Inflation_t$ is the current tobacco inflation.[48] We then calculate RIP following methods used by preceding studies, with a lower affordability index (RIP) indicating that cigarettes have become more affordable and a higher value indicating that cigarettes have become less affordable relative to the reference year.[17 18 44 45] The WHO uses the same approach to obtain its affordability index. Other independent variables used are sex, age, parents', and friends' smoking status, whether offered cigarettes for free, family/class discussion about tobacco, exposure to antismoking messages, and exposure to tobacco advertisements. These variables are selected as they have been shown, theoretically or empirically, to be associated with smoking initiation.[27 31] Except age and RIP, which are continuously measured, all variables are dichotomous.

2.2. Data analysis

We construct a pseudo-longitudinal dataset based on current age and age at first puff. In doing this, we create a historical dataset starting from age 8 (age-at-risk criteria) and follow

For peer review only - http://bmjopen.bmj.com/site/about/guidelines.xhtml

the person until s/he initiates smoking. This was done by inferring the year of first puff using the GYTS question: "How old were you when you first tried a cigarette?" and the age at the time of the survey.[27] STATA routine command, *expand*, is used to expand the person's age at the time of the survey after which an event variable indicating smoking status is created. Students who are below age 8 and who started smoking before age 8 are removed from the dataset. We then link the RIP (affordability index) to this pseudo-longitudinal dataset. Although the GYTS data contain adolescents whose first puff was at age 7 or younger, we assume that a student is at risk at age 8 and exits the sample once smoking is initiated.[10 27 32 33] We choose age 8 as the age-at-risk because that is the age at which the child is relatively developed and able to start out-of-home interaction with peers, according to a previous study.[32] Students who started smoking before reaching age 8 were therefore excluded from the pooled sample and not followed.

Our statistical technique is duration or event history analysis where the timing of transition into initiation is a function of the probability of initiating in period t conditional on not having experienced a transition until period t, known as the hazard rate.[10] Following Nicole Vellios and Corné Van Walbeek [10] and G Emmanuel Guindon, et al. [32], we employ the discrete time-hazard model, with logit specification (see equation 2), to study the association between RIP (affordability) and smoking initiation among adolescents.

$$\Pr(Initiation = 1 \mid X^{'}) = \beta_0 + \beta_1 RIP + \beta_i X^{'} \quad \ldots (2)$$

where Initiation is defined as first cigarette puff, RIP is the affordability index, X' is a vector of other independent variables affecting smoking initiation among adolescents, and $\beta$is a vector of the regression coefficients. The predictors, X' , represent age, sex, whether offered free cigarette, parental and peer smoking status, family/class discussion on the dangers of tobacco, exposure to tobacco advertisements, and hearing of antismoking messages, and

For peer review only - http://bmjopen.bmj.com/site/about/guidelines.xhtml

awareness of tobacco control policies introduced in 2012 (for the 2017 GYTS). We report odds ratios (ORs), and the statistical level of significance is set at $p \leq 0.1$. OR <1 implies that when a higher share of income is required to buy 2000 cigarettes (cigarettes are less affordable), the risk of smoking initiation declines, and vice versa. The partial derivative of equation 2 with respect to RIP gives the affordability elasticity.

To check the robustness of the logistic regression estimates, we employ a propensity score matching (PSM) technique to match ever-smokers to never-smokers based on the propensity scores. Our approach to matching follows previous studies.[33 49 50] The propensity scores are obtained by running a logit regression to estimate the probability of being a smoker based on the variables in equation 2, except RIP, and the predicted probabilities are then used to match ever-smokers to never-smokers. Using the neighborhood matching, ever-smokers are matched to their two nearest neighbors. After matching the sample, we re-estimate the logit model to assess the effect of affordability on the probability of initiating smoking, using GYTS weights on the matched sample.[49 50] With the matching approach, we are able to obtain the effect of affordability on the probability of initiating smoking among adolescent smokers and non-smokers who possess similar characteristics based on the propensity scores. This technique addresses issues of endogeneity and concerns relating to the fact that some never-smokers will never choose to smoke or use any form of tobacco irrespective of market conditions.[33 34] Further, we minimize the problem of endogeneity by not using self-reported prices.[32] Data analysis is conducted using STATA version 15. The study benefited immensely from discrete time modelling guidelines and STATA code produced by Professor Stephen Jenkins.[51]

2.3 Interpretation of RIP Elasticity

For peer review only - http://bmjopen.bmj.com/site/about/guidelines.xhtml

Although the RIP is measured in percentages, interpretation of the affordability index follows the same procedure for elasticity interpretation. The elasticity measures the percentage change in probability of initiating smoking following a percentage change in RIP, ceteris paribus. Assuming the current RIP is 6% or 0.06, then a 1% increase in RIP corresponds to the current RIP increasing from 6% to 6.06%. When using a unit change interpretation, a unit change will be RIP moving from 6% to 7% and therefore probabilities will change in absolute units and not percentages. Such distinction is important in understanding the impact of affordability on smoking behavior.

Patient and Public Involvement:

No patient involved.

3. RESULTS

3.1. Descriptive statistics

A total of 20,202 adolescents were interviewed across the three GYTS waves (2000 – 2009). 54% of the respondents were males, while 76.47% were aged 15 years or less. In the 2017 GYTS, 5,664 people were interviewed, with about 48% being males. Overall, 12.46% and 8.9% of the respondents in the pooled (2000 – 2009) and 2017 surveys, respectively, had ever smoked.

Given our age-at-risk criteria, 15,861 (2000 – 2009 GYTS) and 5,389 (2017 GYTS) people were eligible for inclusion in our pseudo-longitudinal analysis. For surveys prior to 2017, 4.2% initiated smoking at some point between 1991 and 2009, and about 77% of smoking initiators did so before reaching age 16. Further, 67% of initiators were males. Overall, males represented 53.62% of the eligible respondents. In the 2017 survey, 4.72% of the respondents

For peer review only - http://bmjopen.bmj.com/site/about/guidelines.xhtml

initiated smoking at some point between 2008 and 2017. The characteristics of the samples are presented in Table 1. Due to incomplete information on some of the variables, the number of people used in the regression varies.

Table 1: Descriptive statistics

Variable	2000 – 2009 GYTS	2017 GYTS
	Students, n = 15 861	n=5389
Initiated smoking during the period	4.20%	4.72%
RIP (Affordability)	19.87 (SD=6.53)	7.63 (SD =0.86)
Offered free cigarettes	12.44%	8.13%
Sex (male =1)	53.62%	48.73%
At least one parent smoke	11.78%	-
Family/class discuss about tobacco	72.50%	51.47%
At least a friend smoke	15.94%	-
Exposed to tobacco adverts	40.46%	56.03%
Age (Years)	14.15 (SD=1.7)	14.10 (SD =1.03)
Heard anti-smoking campaigns	74.64%	57.26%
Age at initiation (Years)	11.95 (SD = 2.9)	11.26 (SD=2.41)
Percentage of initiators before age 16	77%	94%
Percentage of initiators who are males	67%	59%
Awareness of smoke free policies	-	78.24%

For peer review only

3.2. Regression Results

Results from the logit regressions for the unmatched and matched samples are reported in Tables 2 (GYTS 2000 -2009) and 3 (GYTS 2017). The results show a statistically significant and negative relationship between RIP and smoking initiation. For instance, the results for the full unmatched sample (model 1, Table 2) show that OR on RIP is 0.98. This implies that a unit increase in affordability is associated with 0.98 odds of initiating smoking (OR = 0.98, $p<0.05$). Thus, an adolescent who is subjected to a unit increase in RIP has 2% (i.e., 1 – 0.98)

For peer review only - http://bmjopen.bmj.com/site/about/guidelines.xhtml

× 100 = 2%)) lower probability of initiating smoking than his/her counterpart who is not subjected to the same increase. Note that these results are not elasticities. Females have 40.1% (i.e., 1 – 0.599) × 100 = 40.1%)) lower probability of initiating smoking (OR = 0.599, p<0.01) compared to their male counterparts in the unmatched sample.

Table 2: Effect of RIP on smoking initiation among adolescents (GYTS 2000 -2009)

	Unmatched	Matched
VARIABLES	Odds ratio	Odds ratio
Affordability (RIP)	0.981**	0.974***
	(0.009)	(0.009)
Offered free cigarette (Ref=no)	1.491***	0.517***
	(0.216)	(0.071)
Sex (Ref = male)	0.599***	0.615***
	(0.072)	(0.076)
At least one parent smokes (Ref =no)	2.131***	0.862
	(0.280)	(0.104)
Family/class discussion (Ref = no)	1.001	1.711***
	(0.133)	(0.230)
At least one friend smokes (Ref=no)	4.109***	1.094
	(0.520)	(0.126)
Exposure to adverts (Ref=no)	1.155	1.027
	(0.140)	(0.121)
Age	1.150***	0.991
	(0.042)	(0.031)
Heard of anti-smoking message/campaign (Ref=no)	1.342*	2.048***
	(0.217)	(0.321)
Survey cycle (ref=2000)		
2006	0.958	0.880
	(0.146)	(0.138)
2009	1.108	1.003
	(0.171)	(0.159)
Log (time)	1.110	1.393***
	(0.106)	(0.146)
Constant	0.000***	0.048***
	(0.000)	(0.024)
Observations	106,673	10,078
Number of people	15,201	1,611

For peer review only - http://bmjopen.bmj.com/site/about/guidelines.xhtml

Ever-smokers	611	611
Pseudo R-squared	0.0815	0.0448
Chi2	439.2***	91.84***

Robust standard errors in parentheses
*** p<0.01, ** p<0.05, * p<0.1

Table 3: **Effect of RIP on smoking initiation among adolescents** (GYTS 2017)

	Unmatched	Matched
VARIABLES	Odds ratio	Odds ratio
Affordability (RIP)	0.821**	0.804***
	(0.066)	(0.065)
Sex (Ref=male)	0.659**	0.902
	(0.120)	(0.177)
Offered free cigarettes (Ref= no)	3.403***	0.978
	(0.726)	(0.221)
Heard of anti-smoking message (Ref=no)	1.165	1.009
	(0.213)	(0.192)
Exposed to tobacco adverts (Ref= no)	3.030***	1.893***
	(0.622)	(0.421)
Smoke free policies awareness (Ref=no)	1.250	1.160
	(0.329)	(0.332)
Age	1.847***	1.793***
	(0.294)	(0.279)
Class discussion on tobacco harms (Ref=no)	0.795	1.278
	(0.138)	(0.241)
Log(time)	0.104***	0.119***
	(0.067)	(0.076)
Constant	0.000***	0.002***
	(0.000)	(0.002)
Observations	37,654	4,850
Number of people	5,301	747
Ever-smokers	231	206
Pseudo R-squared	0.0599	0.0292
Chi2	158***	38.72***

Robust standard errors in parentheses
*** p<0.01, ** p<0.05, * p<0.1

For peer review only - http://bmjopen.bmj.com/site/about/guidelines.xhtml

Similarly, in Table 3, in the unmatched sample, an adolescent faced with a unit increase in RIP has about 18% (i.e., 1 – 0.821) × 100 = 17.9%)) lower probability of starting smoking than his/her counterpart who is not subjected to the same increase.

Other significant factors that influence smoking initiation in our two samples include whether the adolescent's parents (OR = 2.131, p<0.01) or friends (OR = 4.109, p<0.01) smoke. In addition, adolescents who are offered free cigarettes have a high probability of initiating smoking (OR = 1.491, p<0.01) compared to those who receive no such offer for the 2000 - 2009 wave (Table 2). In the 2017 wave, the odds of adolescents starting smoking when given cigarettes freely is 3.403 (p<0.01) (Table 3).

In the matched sample (model 2, Table 2), 611 ever-smokers were matched to their two nearest neighbors (1000 never-smokers) which produced a sample of 1,611 adolescents with similar characteristics. An adolescent subjected to a unit increase in RIP has a 0.97 times lower chance of initiating smoking (OR = 0.974, p<0.01) compared to those not exposed to the same increase in RIP. Similarly in the matched sample of the GYTS 2017 (Table 3), a unit increase in RIP is associated with 0.80 times lower odds of smoking initiation (OR = 0.804, p<0.01).

Getting free cigarettes (OR = 0.517, p<0.01), family/class discussion on tobacco (OR = 1.711, p<0.01), and hearing antismoking messages (OR = 2.048, p<0.01) are all found to be statistically significant in influencing smoking initiation in the matched sample (Table 2). However, the odds for these variables are contrary to *a priori* expectations. Similarly, in the matched sample (Table 2), the likelihood of initiating smoking among females is lower. The results imply that females have about 39% lower probability of initiating smoking (OR = 0.615, p<0.01) than males.

3.3. Affordability Elasticities

For peer review only - http://bmjopen.bmj.com/site/about/guidelines.xhtml

In the unmatched sample, the estimated affordability elasticity is -0.372 [CI: -0.701 to -0.042] for the 2000-2009 sample and -1.247 [-2.248 to -0.246] for the 2017 sample. These elasticities are statistically significant at the 5% level. By sex, the affordability elasticity is -0.137 for males and -0.888 for females for the 2000-2009 sample, but only that of females is statistically significant (p<0.01).. The elasticities are higher for 2017 GYTS (-0.938 for males and -1.610 for females). The results are presented in Table 4.

Table4: Affordability Elasticity estimates

	Both sexes		Male		Female	
Panel A: 2000 -2009						
	Percentage changes (d(lny)/d(lnx))					
VARIABLES	Unmatched	Matched	Unmatched	Matched	Unmatched	Matched
Affordability	-0.372**	-0.490***	-0.137	-0.326	-0.888***	-0.928***
	(0.168)	(0.168)	(0.219)	(0.216)	(0.253)	(0.258)
95% CI	-0.701 to -0.042	-0.818 to -0.161	-0.567 to 0.292	-0.749 to 0.097	-1.384 to -0.392	-1.434 to -0.422
Observations	106,673	10,078	55,396	5,648	51,277	4,430
Panel B: 2017 GYTS						
Affordability	-1.247**	-1.349***	-0.938**	-1.045**	-1.610*	1.518**
	(0.511)	(0.500)	(0.474)	(0.484)	(0.866)	(0.778)
95% CI	-2.248 to -0.246	-2.328 to -0.369	-1.867 to -0.008	-1.993 to -0.096	-3.307 to -0.087	-3.043 to -0.007
Observations	37,654	4,850	18,084	2,807	19,570	2,043

Standard errors in parentheses
*** p<0.01, ** p<0.05, * p<0.1

In the matched sample for the 2000-2009 GYTS, the overall elasticity is -0.490 [CI: -0.818 to -0.161] for both sexes (Table 4), which is similar to that of the unmatched sample. For males, the effect of changes in RIP is statistically insignificant. Among females, a percentage increase in RIP is associated with a 0.928% lower probability of smoking initiation. The elasticities for both males and females in the 2017 GYTS were negative, statistically significant, and more than unity.

For peer review only - http://bmjopen.bmj.com/site/about/guidelines.xhtml

The mean and median standardized difference for the covariates used in matching show that the matching satisfies the balancing test (results not reported). The mean and median standardized difference is 2.3%, which is below the normal 10% threshold. Therefore, the balancing property is satisfied.

5. DISCUSSION AND CONCLUSION

In this study, increasing the RIP of cigarettes is significantly associated with a lower probability of initiating smoking. This finding is consistent with international literature, including the few existing studies in SSA that have reported that making cigarettes less affordable lowers the likelihood of smoking initiation among young people.[17 18 26] In addition, the results from the unmatched 2000-2009 sample suggest that males are not responsive to changes in RIP whilst females are. Nevertheless, in the matched sample analysis, especially using the 2017 GYTS, both males and females are responsive to changes in RIP. Indeed, the issue of affordability becomes more important given that Ghana's per capita income has been growing at an average of 4.4% annually in the last decade.[30]

Parental smoking increased the odds of smoking initiating. This points to the parental influence on the lifestyle of adolescents. Adolescents whose parents smoke may perceive smoking as acceptable behavior. Previous studies have reported similar findings.[10 27 31 52] The odds of smoking initiation is higher for those whose friends' smoke. This points to the influence of peers, and is consistent, for example, with Kwok-Kei Mak, et al. [31]. Those who were offered free cigarettes by sales representatives were more likely to initiate smoking. All forms of tobacco promotion and advertising were banned in Ghana in 1982. However, the tobacco industry seems to be breaking these laws, because 12.44% of youth reported being offered a cigarette for free. This observation, together with our results, suggest the need to

For peer review only - http://bmjopen.bmj.com/site/about/guidelines.xhtml

strengthen the enforcement of the existing ban on all forms of tobacco advertising and promotion in Ghana.

This study has several limitations. The GYTS is a self-reporting survey, which means the responses are prone to recall bias even in cases where the adolescents are required to answer questions about events that occurred in the past 30 days. For instance, students may not recall the exact age at which they tried their first puff. There is also a social desirability bias when self-reporting behaviors such as smoking, especially among females. The weakness of our measure of smoking initiation is that it may not predict regular smoking behavior well.[41 42] In addition, there are other important factors affecting smoking uptake among the youth that are not included in this study. For instance, changing community norms regarding smoking, the enforcement of laws regarding the sale of cigarettes to minors, and changes in the social image of smoking are key factors that may influence smoking participation,[53 54] but are not included in the models estimated.

In conclusion, making cigarettes less affordable is associated with a lower probability of smoking initiation among adolescents in Ghana. This supports the use of price measures, through higher excise taxes, as effective strategies to decrease smoking initiation among adolescents in Ghana. Since incomes are rising at the average of 4.4% annually[30], tobacco taxes need to be adjusted regularly to ensure that cigarettes or other tobacco products become less affordable over time in order to discourage young people from initiating smoking and to encourage smokers to quit.

Author Contributions: Conceptualization, M.K.B.; methodology, M.K.B; software, M.K.B.; validation, M.K.B.., M.I., E.N.T., N.D.M., and H.R.; formal analysis, M.K.B.; investigation, M.K.B., M.I., E.N.T., N.D.M., and H.R.; data curation, M.K.B; writing—original draft preparation, M.K.B., M.I., E.N.T., N.D.M., and H.R.; writing—review and editing, M.K.B., M.I., E.N.T., N.D.M., H.R.; project administration, M.K.B.; supervision, H.R.; funding acquisition, H.R. All authors have read and approved the manuscript.

For peer review only - http://bmjopen.bmj.com/site/about/guidelines.xhtml

Funding: Funding for this study was provided by Cancer Research UK (Grant number: C62640/A24723) and International Development Research Centre (Grant Number: 108820-001), with additional support from the SAMRC/Wits Centre for Health Economics and Decision Science – PRICELESS SA (grant number 23108). Funding for ENT and NDM is acknowledged from the Tobacco Control Capacity Programme (Grant Number: MR/P027946/2) supported by UK Research and Innovation (UKRI) with funding from the Global Challenges Research Fund (GCRF). The funders had no role in study design, data collection and analysis, or preparation of the manuscript.

Conflicts of Interest: The authors declare that they have no conflict of interest.

Research Ethics Approval: Human Participants

This study does not involve human participants.

Research Ethics Approval: Animals

This study does not involve animal subjects.

Patient consent for publication: No patient involved.

Data Availability statement: The publicly available data can be accessed: https://nccd.cdc.gov/GTSSDataSurveyResources/Ancillary/DataReports.aspx?CAID=2. The relative income price data are available from the authors on reasonable request.

REFERENCES

1. Doll R, Peto R, Boreham J, et al. Mortality in relation to smoking: 50 years' observations on male British doctors. *Bmj* 2004;328(7455):1519.

2. Winkler V, Mangolo NJ, Becher H. Lung cancer in South Africa: a forecast to 2025 based on smoking prevalence data. *BMJ open* 2015;5(3):e006993.

For peer review only - http://bmjopen.bmj.com/site/about/guidelines.xhtml

3. World Health Organization. Tobacco: Key Facts. 2020 [updated 27 May 2020. Available from: https://www.who.int/news-room/fact-sheets/detail/tobacco accessed 22 November 2020.

4. Goodchild M, Nargis N, d'Espaignet ET. Global economic cost of smoking-attributable diseases. *Tobacco Control* 2018;27(1):58-64.

5. Boachie MK, Rossouw L, Ross H. The Economic Cost of Smoking in South Africa, 2016. *Nicotine Tob Res* 2021;23(2):286 - 93. doi: 10.1093/ntr/ntaa162

6. USDHHS. Preventing tobacco use among youth and
young adults: A report of the surgeon general 2012 [Available from: http://www.surgeongeneral.gov/library/reports/preventing-youth-tobacco-use/full-report.pdf (accessed: Jan 3, 2019).

7. World Health Organization. WHO global report on trends in prevalence of tobacco use 2000-2025. 3rd ed. Geneva: World Health Organization, 2019.

8. CDC, WHO, MOH. GLOBAL YOUTH TOBACCO SURVEY: Factsheet, Ghana 2017, 2018.

9. World Health Organization. WHO global report on trends in prevalence of tobacco smoking 2015. Geneva: World Health Organization, 2015.

10. Vellios N, Van Walbeek C. Determinants of regular smoking onset in South Africa using duration analysis. *BMJ Open* 2016;6(7):e011076.

11. IARC. Effectiveness of tax and price policies for tobacco control. In: Cancer. IAfRo, ed. USA: International Agency for Research on Cancer, 2011.

12. Boachie MK, Ross H. Determinants of smoking intensity in South Africa: evidence from township communities. *Preventive Medicine Reports* 2020;19:101099.

13. Van Walbeek C. A simulation model to predict the fiscal and public health impact of a change in cigarette excise taxes. *Tobacco control* 2010;19(1):31-36.

14. Yurekli A, de Beye J. Design and administer tobacco taxes. *World Bank economics of tobacco toolkit* 2001(4)

15. Van Walbeek C. Raising Additional Government Revenues in Ghana by Raising the Excise Tax on Tobacco and Alcohol Washington, D.C.: WBG Global Tobacco Control

For peer review only - http://bmjopen.bmj.com/site/about/guidelines.xhtml

Program, World Bank Group; 2014 [Available from: http://documents.worldbank.org/curated/en/133561561707742558/pdf/Raising-Additional-Government-Revenues-in-Ghana-by-Raising-the-Excise-Tax-on-Tobacco-and-Alcohol.pdf accessed 15 July 2019.

16. He Y, Shang C, Chaloupka FJ. The association between cigarette affordability and consumption: An update. *PLOS ONE* 2018;13(12):e0200665. doi: 10.1371/journal.pone.0200665

17. Nargis N, Stoklosa M, Shang C, et al. Price, Income, and Affordability as the Determinants of Tobacco Consumption: A Practitioner's Guide to Tobacco Taxation. *Nicotine and Tobacco Research* 2021;23(1):40-47.

18. Blecher E, Van Walbeek C. An international analysis of cigarette affordability. *Tobacco Control* 2004;13(4):339-46.

19. Ding A. Youth are more sensitive to price changes in cigarettes than adults. *The Yale journal of biology and medicine* 2003;76(3):115.

20. Ho L-M, Schafferer C, Lee J-M, et al. The effect of cigarette price increases on cigarette consumption, tax revenue, and smoking-related death in Africa from 1999 to 2013. *International journal of public health* 2017;62(8):899-909.

21. Chelwa G, van Walbeek C. Does cigarette demand respond to price increases in Uganda? Price elasticity estimates using the Uganda National Panel Survey and Deaton's method. *BMJ open* 2019;9(3):e026150. doi: 10.1136/bmjopen-2018-026150 [published Online First: 2019/03/23]

22. Stoklosa M, Goma F, Nargis N, et al. Price, tax and tobacco product substitution in Zambia: findings from the ITC Zambia Surveys. *Tob Control* 2019;28(Suppl 1):s45-s52. doi: 10.1136/tobaccocontrol-2017-054037 [published Online First: 2018/03/27]

23. Mukong AK, Tingum EN. The demand for cigarettes: New evidence from South Africa. *Development Southern Africa* 2020;37(1):40-54.

24. Tingum EN, Mukong AK, Mdege N. The effects of price and non-price policies on cigarette consumption in South Africa. *Tob Induc Dis* 2020;18(July) doi: 10.18332/tid/123424

For peer review only - http://bmjopen.bmj.com/site/about/guidelines.xhtml

25. Kidane A, Mduma J, Naho A, et al. The Demand for Cigarettes in Tanzania and Implications for Tobacco Taxation Policy. *Advances in economics and business* 2015;3(10):428.

26. Kostova D. A (nearly) global look at the dynamics of youth smoking initiation and cessation: the role of cigarette prices. *Applied Economics* 2013;45(28):3943-51.

27. Asare S, Stoklosa M, Drope J, et al. Effects of Prices on Youth Cigarette Smoking and Tobacco Use Initiation in Ghana and Nigeria. *International Journal of Environmental Research and Public Health* 2019;16(17):3114.

28. Ministry of Health. Needs assessment for the Implementation of WHO FCTC in Ghana. Accra: Ministry of Health,, 2010.

29. Gov't. of Ghana. Customs and Excise (Duties and Other Taxes) Amendment Act 739. , 2007.

30. World Bank. World Development Indicators.: World Bank; 2019 [Available from: https://databank.worldbank.org/source/world-development-indicators. accessed 01 Jan 2020.

31. Mak K-K, Ho S-Y, Day JR. Smoking of parents and best friend—independent and combined effects on adolescent smoking and intention to initiate and quit smoking. *Nicotine Tob Res* 2012;14(9):1057-64.

32. Guindon GE, Paraje GR, Chávez R. Prices, inflation, and smoking onset: the case of Argentina. *Economic Inquiry* 2018;56(1):424-45.

33. Dauchy E, Ross H. The Effect of Price and Tax Policies on the Decision to Smoke among Men in Kenya. *Addiction* 2019;114(7):1249-63.

34. Li M. Using the propensity score method to estimate causal effects: A review and practical guide. *Organizational Research Methods* 2013;16(2):188-226.

35. Warren CW. Tobacco use among youth: a cross country comparison. *Tobacco Control* 2002;11(3):252-70.

36. Gov't. of Ghana. Public Health Act, 2012 Act 851. Ghana 2012.

37. Wellington E. Ghana Global Youth Tobacco Survey

For peer review only - http://bmjopen.bmj.com/site/about/guidelines.xhtml

(GYTS), Factsheet. 2006 [Available from: http://www.afro.who.int/en/downloads/docdownload/1887-ghana-gyts-2006-factsheet.html (Retrieved Jan 28, 2019).

38. Cadmus EO, Ayo-Yusuf OA. The effect of smokeless tobacco use and exposure to cigarette promotions on smoking intention among youths in Ghana. *Cogent Medicine* 2018;5(1):1531459.

39. CDC and WHO. Ghana global youth tobacco survey. Atlanta, United States: Centers for Disease Control and Prevention (CDC). 2006.

40. Freedman KS, Nelson NM, Feldman LL. Smoking initiation among young adults in the United States and Canada, 1998-2010: a systematic review. *Preventing chronic disease* 2012;9

41. Azagba S, Baskerville NB, Minaker L. A comparison of adolescent smoking initiation measures on predicting future smoking behavior. *Preventive medicine reports* 2015;2:174-77.

42. Reidpath DD, Davey TM, Kadirvelu A, et al. Does one cigarette make an adolescent smoker, and is it influenced by age and age of smoking initiation? Evidence of association from the US Youth Risk Behavior Surveillance System (2011). *Preventive medicine* 2014;59:37-41.

43. Centers for Disease Control Prevention. Selected cigarette smoking initiation and quitting behaviors among high school students--United States, 1997. *MMWR Morbidity and mortality weekly report* 1998;47(19):386.

44. Blecher E. Targeting the affordability of cigarettes: a new benchmark for taxation policy in low-income and-middle-income countries. *Tobacco control* 2010;19(4):325-30.

45. Nargis N, Stoklosa M, Drope J, et al. Trend in the affordability of tobacco products in Bangladesh: findings from the ITC Bangladesh Surveys. *Tobacco control* 2019;28(Suppl 1):s20-s30.

46. Philip Morris International. World summary of cigarette retail prices, tax burdens and tax incidence.: Philips Moris Records 1996 [Available from: https://www.industrydocuments.ucsf.edu/docs/#id=glhf0066.

For peer review only - http://bmjopen.bmj.com/site/about/guidelines.xhtml

47. Ghana Gto. Report card on the WHO Framework Convention on Tobacco Control., 2009.

48. Ghana Statistical Service. Consumer Price Index (CPI): Time Series In: Service GS, ed. Accra, 2012.

49. Austin PC, Jembere N, Chiu M. Propensity score matching and complex surveys. *Statistical methods in medical research* 2018;27(4):1240-57.

50. DuGoff EH, Schuler M, Stuart EA. Generalizing observational study results: applying propensity score methods to complex surveys. *Health services research* 2014;49(1):284-303.

51. Jenkins SP. Survival Analysis with Stata. [Available from: https://www.iser.essex.ac.uk/resources/survival-analysis-with-stata accessed 01 January 2020.

52. Gilman SE, Rende R, Boergers J, et al. Parental smoking and adolescent smoking initiation: an intergenerational perspective on tobacco control. *Pediatrics* 2009;123(2):e274-e81.

53. Gilpin EA, Pierce JP. Trends in adolescent smoking initiation in the United States: is tobacco marketing an influence? *Tobacco Control* 1997;6(2):122-27.

54. Cawley J, Markowitz S, Tauras J. Obesity, cigarette prices, youth access laws and adolescent smoking initiation. *Eastern Economic Journal* 2006;32(1):149-70.

For peer review only

For peer review only - http://bmjopen.bmj.com/site/about/guidelines.xhtml

STROBE Statement—Checklist of items that should be included in reports of ***cross-sectional studies***

	Item No	Recommendation	Page No
**Title and abstract**	1	(*a*) Indicate the study's design with a commonly used term in the title or the abstract	1
		(*b*) Provide in the abstract an informative and balanced summary of what was done and what was found	2
**Introduction**			
Background/rationale	2	Explain the scientific background and rationale for the investigation being reported	5-7
Objectives	3	State specific objectives, including any prespecified hypotheses	6
**Methods**			
Study design	4	Present key elements of study design early in the paper	7
Setting	5	Describe the setting, locations, and relevant dates, including periods of recruitment, exposure, follow-up, and data collection	7-8
Participants	6	(*a*) Give the eligibility criteria, and the sources and methods of selection of participants	7-9
Variables	7	Clearly define all outcomes, exposures, predictors, potential confounders, and effect modifiers. Give diagnostic criteria, if applicable	8-9
Data sources/ measurement	8*	For each variable of interest, give sources of data and details of methods of assessment (measurement). Describe comparability of assessment methods if there is more than one group	8-9
Bias	9	Describe any efforts to address potential sources of bias	10
Study size	10	Explain how the study size was arrived at	8
Quantitative variables	11	Explain how quantitative variables were handled in the analyses. If applicable, describe which groupings were chosen and why	9
Statistical methods	12	(*a*) Describe all statistical methods, including those used to control for confounding	9-10
		(*b*) Describe any methods used to examine subgroups and interactions	9-10
		(*c*) Explain how missing data were addressed	9-10
		(*d*) If applicable, describe analytical methods taking account of sampling strategy	9-10
		(*e*) Describe any sensitivity analyses	11
**Results**			
Participants	13*	(a) Report numbers of individuals at each stage of study—eg numbers potentially eligible, examined for eligibility, confirmed eligible, included in the study, completing follow-up, and analysed	12
		(b) Give reasons for non-participation at each stage	
		(c) Consider use of a flow diagram	
Descriptive data	14*	(a) Give characteristics of study participants (eg demographic, clinical, social) and information on exposures and potential confounders	13
		(b) Indicate number of participants with missing data for each variable of interest	
Outcome data	15*	Report numbers of outcome events or summary measures	
Main results	16	(*a*) Give unadjusted estimates and, if applicable, confounder-adjusted estimates and their precision (eg, 95% confidence interval). Make clear which confounders were adjusted for and why they were included	13-17

For peer review only - http://bmjopen.bmj.com/site/about/guidelines.xhtml

		(*b*) Report category boundaries when continuous variables were categorized	
		(*c*) If relevant, consider translating estimates of relative risk into absolute risk for a meaningful time period	
Other analyses	17	Report other analyses done—eg analyses of subgroups and interactions, and sensitivity analyses	
**Discussion**			
Key results	18	Summarise key results with reference to study objectives	18-19
Limitations	19	Discuss limitations of the study, taking into account sources of potential bias or imprecision. Discuss both direction and magnitude of any potential bias	19
Interpretation	20	Give a cautious overall interpretation of results considering objectives, limitations, multiplicity of analyses, results from similar studies, and other relevant evidence	11-12
Generalisability	21	Discuss the generalisability (external validity) of the study results	19
**Other information**			
Funding	22	Give the source of funding and the role of the funders for the present study and, if applicable, for the original study on which the present article is based	20

*Give information separately for exposed and unexposed groups.

Note: An Explanation and Elaboration article discusses each checklist item and gives methodological background and published examples of transparent reporting. The STROBE checklist is best used in conjunction with this article (freely available on the Web sites of PLoS Medicine at http://www.plosmedicine.org/, Annals of Internal Medicine at http://www.annals.org/, and Epidemiology at http://www.epidem.com/). Information on the STROBE Initiative is available at www.strobe-statement.org.

For peer review only

For peer review only - http://bmjopen.bmj.com/site/about/guidelines.xhtml

Effect ofThe association between relative income price onand smoking initiation among adolescents in Ghana: Evidence from pseudo-longitudinal data

Micheal Kofi Boachie[1, 2, 3], Mustapha Immurana[34], Ernest N. Tingum[2, 45], Noreen Dadirai Mdege[56], Hana Ross[2]**

[1] SAMRC/Wits Centre for Health Economics and Decision Science -PRICELESS SA, School of Public Health, University of the Witwatersrand, Johannesburg 2193, South Africa. Department of Health Policy Planning and Management, School of Public Health, University of Health and Allied Sciences, Ho, Ghana; mkboachie@gmail.com

[2] Research Unit on the Economics of Excisable Products, School of Economics, University of Cape Town, Cape Town, South Africa; hana.ross@uct.ac.za

[3] SAMRC Centre for Health Economics and Decision Science, School of Public Health, University of the Witwatersrand, Johannesburg 2193, South Africa.

[43] Institute of Health Research, University of Health and Allied Sciences, Ho, Ghana; mimmurna@uhas.edu.gh; mustaphaimmurana@gmail.com

[54] Department of Economics, University of Namibia, Windhoek, Namibia; ngehernest@gmail.com

[65] Department of Health Sciences, University of York, Heslington, United Kingdom; noreen.mdege@york.ac.uk

** Correspondence: mkboachie@gmail.com; Tel.: +27 845 644 650233 50 258 9141.

Word count:

Abstract: 236

Introduction: 678

Materials and Methods: 1191

Results: 648

Discussion and Conclusion: 464

Total word count: 2952

Number of References: 47

Abstract: 290

1

For peer review only

Introduction: 741

Materials and Methods: 1517

Results: 1330

Discussion and Conclusion: 508

Total word count: 4386

Number of References: 54

For peer review only

Abstract:

Objective: Many smokers initiate smoking during adolescence. Making tobacco products less affordable is one of the best ways to control tobacco use. Studies on the effect of relative income price (RIP (i.e., affordability)) of cigarettes on smoking initiation are scarce in low- and middle-income countries, especially in Sub-Saharan Africa where data are limited. The goal of this study is to examine the effect of cigarette RIP on adolescent smoking initiation in Ghana.

Setting: The study uses a pseudo-longitudinal dataset constructed from the Global Youth Tobacco Surveys (GYTS (2000-2009 and 2017)) and RIP for the most sold cigarette brand in Ghana.

Participants: The GYTS is a national survey on adolescents.

Primary and secondary outcome: Effect of RIP on adolescent smoking initiation in Ghana.

Results: We find that the probability of smoking initiation falls significantly in response to a higher RIP, with an elasticity of -0.374 (95% CI: -0.692 to -0.057) for the overall or

~~unmatched sample and -0.547 (95% CI: -0.868 to -0.226) for the matched sample. The RIP elasticity for females (-0.685) (95% CI: -1.221 to -0.149) is statistically significant at 5% in the unmatched sample. In the matched sample, the elasticity is statistically significant at 1% for both males (-0.544) (95% CI: -0.864 to -0.224) and females (-0.551) (95% CI: -0.874 to -0.229).~~

Using the GYTS 2000-2009 data, ~~W~~we find that the probability of smoking initiation falls significantly in response to a higher RIP, with an elasticity of -0.372 (95% CI: -0.701 to -0.042) for the ~~2000-2009~~ unmatched sample and -0.490 (95% CI: -0.818 to -0.161) for the matched sample. The RIP elasticity for females [(-0.888) (95% CI: --1.384 to -0.392) and (-0.928) (95% CI: -1.434 to -0.422)] is statistically significant at 1% in both the unmatched and the matched samples, respectively, while the RIP elasticity for males ~~for while t~~~~was~~is statistically insignificant in the 2000 - 2009 surveys. Analysis of the 2017 GYTS shows a similar outcome: a negative relationship between RIP and smoking initiation, and the results are statistically significant for both male and female, and for both matched and unmatched samples.

Conclusion: The affordability (RIP) of cigarettes is negatively related to the probability of smoking initiation among adolescents in Ghana. Raising tobacco taxes in line with income growth would make cigarettes less affordable and dissuade adolescents from initiating smoking.

Keywords: prices; taxes; smoking initiation; adolescents; relative income price

For peer review only

Strengths and Limitations of the study

- Research shows that higher cigarette prices reduce smoking and that making cigarettes less affordable is one of the best ways to control tobacco use.

- This is the first study to explore the impact of Relative Income Price (i.e., affordability index) of cigarettes on youth smoking initiation in Sub-Saharan Africa.
- Our study addresses the evidence gap on tThe impact of cigarette prices on smoking initiation is still being debated. Only few studies have investigated the impact of cigarette relative income price (RIP), a proxy for their affordability, on smoking initiation . There is no such study using data fromin Sub-Saharan Africa.
- Our analysis controls for variables that are known, empirically or theoretically, to be associated with smoking initiation, and the relationship is tested using a pseudo-longitudinal dataset of 17 years. We examined the link between cigarette RIP and adolescent smoking initiation in Ghana and found an inverse relationship. This means that cigarette prices need to grow faster than inflation and income in order for them to have a negative impact on smoking uptake.
- We also examine potential sex differences in the effect of affordability on cigarette smoking initiation: this is key to the implementation of tobacco control policies that confer adequate protection across both genders.
- Data for our smoking initiation variable is from self-report.

The findings may not be generalized to other countries since it uses a single country data. Since GYTS data are available in many low- and middle-income countries (LMIC), our study provide a template on how to do analyses elsewhere in order to enhance our understating of the impact of cigarette affordability on smoking uptake in LMIC.

For peer review only - http://bmjopen.bmj.com/site/about/guidelines.xhtml

- The results are subject to self-reporting and recall biases as well as omitted variable bias due to lack of data on other factors affecting smoking uptake.

For peer review only

1. INTRODUCTION

Tobacco use is one of the major risk factors for many non-communicable diseases such as lung cancer and ischemic heart disease,[1 2] and it accounts for over eight million deaths annually worldwide.[3] Tobacco use also imposes huge financial burdens on households and governments.[4 5] The majority (80-90%) of the one billion adult smokers globally began the habit during their adolescence.[6] The current smoking prevalence in Ghana is still low relative to other African countries (3.2% among adults aged 15 years and older,[7] and 6.4% among students aged 13-15 years [8]). However, the number of smokers is predicted to increase from 1.3 million to 1.7 million (i.e., by about 30%) between 2020 and 2025.[9] The expected increase in the number of smokers will be partly driven by initiation among adolescents, therefore lowering initiation is key to slowing down the tobacco epidemic in Ghana.

For peer review only - http://bmjopen.bmj.com/site/about/guidelines.xhtml

Empirical evidence shows that tobacco consumption (initiation and intensity) is significantly inversely related to price.[10-12] In addition to being the most cost-effective measure to reduce tobacco use, increasing the taxes of tobacco products generates revenue for governments.[13-15] Nevertheless, economic factors such as income growth can negatively affect the response of tobacco consumption to tax/price.[16 17] Increasing tobacco prices can be more effective in reducing tobacco consumption if it reduces affordability.[18] Affordability (relative income price (RIP)) elasticity which measures the sensitivity of consumers to real changes in both price and income, can therefore be a useful parameter to explain and predict the sensitivity of consumers to tobacco tax and price policies even in the presence of income growth.[17] This is particularly important for tobacco control measures aimed at adolescents because they are more price sensitive than adults,[12 19] for instance in Ghana where 71.3% of current cigarette smokers aged 13-15 buy their own cigarettes.[8]

Some Sub-Saharan African (SSA) studies, albeit few, have demonstrated that increasing higher cigarette prices reduces smoking prevalence and intensity of use.[12 20-25] However, there is a scarcity of studies on the relationship between cigarette prices and smoking initiation in the SSA context. One study, using data from 48 countries, including four from SSA (Kenya, Nigeria, Senegal, and South Africa), concluded that higher cigarette prices reduce smoking initiation in early youth, with girls being more responsive than boys.[26] However, findings from the SSA countries were not reported separately from the overall study findings. A study in South Africa reported a significant reduction in regular smoking initiation among males due to higher cigarette prices, but not among females [10]. Another study in Nigeria and Ghana concluded that increasing cigarette prices resulted in a reduction in both 30-day cigarette smoking and cigarette smoking onset in both countries.[27]

Ghana has implemented a number of tobacco tax changes over the last 20 years. For example, it introduced a specific excise tax in 2007 and subsequently switched to an ad valorem tax

For peer review only

structure in 2010.[28 29] At the same time, per capita income in Ghana has been growing at an average rate of 4.4% annually in the last decade.[30] These changes have implications for the retail prices of tobacco products (e.g., cigarettes), and the affordability of cigarettes or other tobacco products~~smoking~~. However, to our knowledge, no study has analyzed the impact of cigarette affordability on smoking initiation in Ghana.

We address this critical evidence gap by examining the effect of ~~association between~~ cigarette affordability on~~and~~ smoking initiation among adolescents in Ghana using~~-~~. We hypothesize that making cigarettes less affordable reduces the likelihood of smoking initiation among young people, and ~~while utilis~~make use of~~ing~~, ~~and make use of~~ the Global Youth Tobacco Survey (GYTS) data ~~as well as~~and other datasets to test that hypothesis. The control variables used are sex, age, parents' and friends' smoking status, being offered a cigarette for free, family/class discussion about tobacco, exposure to antismoking messages, and exposure to tobacco advertisements. These controls are based on variables that are known, empirically or theoretically, to be associated with smoking initiation.[27 31]

Our analysis addresses the potential endogeneity of price, if any, as a driver of cigarette demand through~~by~~ i) using aggregate level prices and not self-reported prices[32], ii) ~~and~~ the use of propensity score matching techniques~~,~~[33 34]~~;~~[32] ~~as well as~~ and iii) the fact that some people will never choose to smoke or use any form of tobacco, for example, for reasons of health or religious belief[33 34]~~.~~ We also examine potential sex differences in the ~~association between~~effect of affordability ~~and~~ on cigarette smoking initiation. An understanding of these dynamics is key to the implementation of context-specific tobacco and non-communicable disease control policies in Ghana. ~~We hypothesize that affordability of cigarettes in Ghana is related to smoking initiation among youth Ghana while taking into account sex, age, parents' and friends' smoking status, being offered a cigarette for free, family/class discussion about tobacco, exposure to antismoking messages, and exposure to tobacco advertisements. These~~

For peer review only - http://bmjopen.bmj.com/site/about/guidelines.xhtml

controls are based on variables that are known, empirically or theoretically, to be associated with smoking initiation, including in low- and middle-income countries.[27 31]

2. MATERIALS AND METHODS

2.1. Data and variables

We make use of three waves (2000, 2006 and 2009) of the GYTS lobal Youth Tobacco Survey (GYTS) and RIP (affordability) data (1991–2009) to analyze the effect of affordability on smoking initiation among adolescents in Ghana. The World Health Organization (WHO) defines adolescents as young people between the ages of 10 and 19 years. The GYTS questionnaire specifies ages from 11 years or younger to 17 years or higher for current age (i.e., age at the time of survey). It also captures age at first puff, which ranges from 7 years or younger to 16 years or older. For the purposes of this study, we classify respondents as adolescents, youth or young people. The terms are used interchangeably in the study. The GYTS is a school-based survey developed to enhance the capacity of countries to monitor tobacco use among the youth, as well as implement and evaluate tobacco control and prevention programs.[35] These data provide representative trends of tobacco use among adolescents. The GYTS is a cross-sectional survey and does not follow individuals over time, but provides data on smoking patterns among adolescents. In countries where it is conducted at regular intervals, it allows the monitoring of trends over time. We are aware of the 2017 GYTS for Ghana, but we do not include it in the analysis of the pooled 2000 – 2009 surveys due to inconsistencies in the questions asked, compared to those in previous GYTS surveys. There is no other survey on adolescents in Ghana with comparable measures except the ones outlined. However, wWe analyze the 2017 GYTS separately while linking it withand linked to RIP data for 2008 – 2017 based on age-at-risk criteria (as done for the 2000 – 2009 surveys).

For peer review only - http://bmjopen.bmj.com/site/about/guidelines.xhtml

In Ghana, there was no law prohibiting the sale of cigarettes to minors until ~~minimum age law on smoking until~~ 2012 when restrictions on sale to persons below age 18 years were introduced.[36] ~~t~~The GYTS sample is drawn using a two-stage cluster-sampling design.[35 37 38] Schools are selected with probability proportional to school enrolment size during the first stage, and then classes within participating schools are selected as a systematic equal probability sample with a random start during the second stage. All students in the selected classes are eligible to participate in the survey.

The Ghana GYTS questionnaire captures information on the use of tobacco products such as cigarettes and shisha. The data also include parental and peer smoking habits, perception about tobacco use (e.g., weight gain, health effects, and ease of quitting), amount spent on tobacco in the last 30 days before the survey, and second-hand smoking (SHS).[39] Studies vary widely on the way they define or measure smoking initiation.[40-42] For the GYTS, smoking initiation is measured using the definition of a lifetime smoker, i.e., a person who has ever tried smoking, *even one or two puffs of a cigarette*.[27 42 43] Thus, for our study, smoking initiation (dependent variable) is a dichotomous variable generated from the following GYTS question where students answer Yes/No: *Have you ever tried or experimented with cigarette smoking, even one or two puffs?*.[27]

The main independent variable is the affordability index or the RIP, measured as the percentage of GDP per capita (per capita income) required to buy 100 packs of cigarettes (20 sticks per pack, in total 2,000 sticks)~~100 packs of 2000 cigarettes~~.[17 18 44 45] Affordability is a relative measure and is calculated using nominal prices and nominal GDP per capita, or real prices and real GDP per capita. Data on per capita income are obtained from the World Bank's World Development Indicators,[30] and those of average cigarette prices (for the most-sold brand) come from the WHO [46], ~~and~~ relevant publications of the tobacco industry[47] and

For peer review only

the Government of Ghana[48]. Years with missing data on prices were interpolated using the formula: $P_{t-1} = \frac{P_t}{(1+Tob.Inflation_t)}$ .... (1)

where P_{t-1} is the previous yearsyear's price of cigarette, P_t is current price of cigarette and $Tob.Inflation_t$ is the current tobacco inflation.[49] We then calculate RIP following methods used by preceding studies, with a lower affordability index (RIP) indicating that cigarettes have become more affordable and a higher value indicating that cigarettes have become less affordable relative to the reference year.[17 18 44 45] The WHO uses the same approach to obtain its affordability index. However, the data from WHO cover only the period 2008 – 2018. Other independent variables used are sex, age, parents' and friends' smoking status, whether offered cigarettes for free, family/class discussion about tobacco, exposure to antismoking messages, and exposure to tobacco advertisements. These variables are selected as they have been shown, theoretically or empirically, to be associated with smoking initiation.[27 31] Except age and RIP, which are continuously measured, all variables are dichotomous.

2.2. Data analysis

We construct a pseudo-longitudinal dataset based on current age and age at first puff. In doing this, we create a historical dataset starting from age 8 (age-at-risk criteria) and following the person until s/he initiates smoking. This was done by inferring the year of first puff using the GYTS question: "How old were you when you first tried a cigarette?" and the age at the time of the survey.[27] We then link the RIP (affordability index) to this pseudo-longitudinal dataset. Although the GYTS data contain adolescents whose first puff was at age 7 or younger, we assume that a student is at risk at age 8 and exits the sample once smoking is initiated.[10 27 32 33] We choose age 8 as the age-at-risk because that is the age at which the child is relatively developed and able to start out-of-home interaction with peers, according to

For peer review only

For peer review only - http://bmjopen.bmj.com/site/about/guidelines.xhtml

a previous studyies.[32] Students who started smoking before reaching age 8 were therefore excluded from the pooled sample and not followed.

Our statistical technique is duration or event history analysis where the timing of transition into initiation is a function of the probability of initiating in period *t* conditional on not having experienced a transition until period *t*, known as the hazard rate.[10] Following Nicole Vellios and Corné Van Walbeek [10] and G Emmanuel Guindon, et al. [32], we employ the discrete time-hazard model, with logit specification (see equation 21), to study the association between RIP (affordability) and smoking initiation among adolescents.

$$\Pr(Initiation = 1 \mid X') = \beta_0 + \beta_1 RIP + \beta_i X' \quad \ldots \ (12),$$

where Initiation is defined as first cigarette puff, RIP is the affordability index, X' is a vector of other independent variables affecting smoking initiation among adolescents, and $\beta$is a vector of the regression coefficients. The predictors, X' , represent age, sex, whether offered free cigarette, parental and peer smoking status, family/class discussion on the dangers of tobacco, exposure to tobacco advertisements, and hearing of antismoking messages, and awareness of tobacco control policies introduced in 2012 (for the 2017 GYTS).. We report odds ratios (ORs), and the statistical level of significance is set at p ≤ 0.105. OR <1 implies that when a higher share of income is required to buy 2000 cigarettes (cigarettes are less affordable), the risk of smoking initiation declines, and vice versa. The partial derivative of equation 21 with respect to RIP gives the affordability elasticity.

To check the robustness of the logistic regression estimates, we employ a propensity score matching (PSM) technique to match ever-smokers to never-smokers based on the propensity scores. Our approach to matching follows previous studies.[33 50 51] The propensity scores are obtained by running a logit regression to estimate the probability of being a smoker based on the variables in equation 21, except RIP, and the predicted probabilities are then used to

For peer review only

For peer review only - http://bmjopen.bmj.com/site/about/guidelines.xhtml

match ever-smokers to never-smokers. Using the neighborhood matching, ever-smokers are matched to their two nearest neighbors. After matching the sample, we re-estimate the logit model to assess the effect of affordability on the probability of initiating smoking, using GYTS weights on the matched sample.[50 51] With the matching approach, we are able to obtain the effect of affordability on the probability of initiating smoking among adolescent smokers and non-smokers who possess similar characteristics based on the propensity scores. This technique addresses issues of endogeneity and concerns relating to the fact that some never-smokers will never choose to smoke or use any form of tobacco irrespective of market conditions.[33 34] Further, we minimize the problem of endogeneity by not using self-reported prices.[32] Data analysis is conducted using STATA version 15. The study benefited immensely from discrete time modelling guidelines and STATA code produced by Professor Stephen Jenkins.[52]

For peer review only

2.3 Interpretation of RIP Elasticity

Although the RIP is measured in percentages, interpretation of the affordability index follows the same procedure for elasticity interpretation. The elasticity measures the percentage change in probability of initiating smoking following a percentage change in RIP, ceteris paribus. Assuming the current RIP is 6% or 0.06, then a 1% ~~percentage~~ increase in RIP corresponds to the current RIP increasing from 6% to 6.06%. When using a unit change interpretation, a unit change will be RIP moving from 6% to 7% and therefore probabilities will change in absolute units and not percentages. Such distinction is important in understanding the impact of affordability on smoking behavior.

Patient and Public Involvement:

No patient involved.

For peer review only - http://bmjopen.bmj.com/site/about/guidelines.xhtml

3. RESULTS

3.1. Descriptive statistics

A total of 20,202 adolescents were interviewed across the three GYTS waves (2000 – 2009). 54% of the respondents were males, while 76.47% were aged 15 years or less. In the 2017 GYTS, 5,664 people were interviewed, with about 48% being males. Overall, 12.46% and 8.9% of the respondents in the pooled ~~three surveys~~ (2000 – 2009) and 2017 surveys, respectively, had ever smoked. ~~(tabular results are available on request)~~.

Given our age-at-risk criteria, 15,861 (2000 – 2009 GYTS) and 5,389 (2017 GYTS) people were eligible for inclusion in our pseudo-longitudinal analysis. For surveys prior to 2017~~Of this number~~, 4.2% initiated smoking at some point between 1991 and 2009, and about 77% of smoking initiators did so before reaching age 16. Further, 67% of initiators were males. Overall, males represented 53.62% of the eligible respondents. In the 2017 survey, 4.72% of the respondents' initiated smoking at some point between 2008 and 2017. The characteristics of the samples are presented in Table 1. Due to incomplete information on some of the variables, the number of people used in the regression varies. ~~Characteristics of the sample are shown in Table 1.~~

For peer review only

Table 1: Descriptive statistics

~~Variable~~	~~Students, n = 15 861~~
~~Initiated smoking during the period~~	~~4.20%~~
~~RIP (Affordability)~~	~~19.87 (SD=6.53)~~
~~Offered free cigarettes~~	~~12.44%~~
~~Sex (male =1) sample~~	~~53.62%~~
~~At least one parent smoke~~	~~11.78%~~
~~Family/class discuss about tobacco~~	~~72.50%~~
~~At least a friend smoke~~	~~15.94%~~
~~Exposed to tobacco adverts~~	~~40.46%~~
~~Age (Years)~~	~~14.15 (SD=1.7)~~
~~Heard anti-smoking campaigns~~	~~74.64%~~

Age at initiation (Years)	11.95 (SD = 2.9)
Percentage of initiators before age 16	77%
Percentage of initiators who are males	67%

Variable	2000 – 2009 GYTS	2017 GYTS
	Students, n = 15 861	n=5389
Initiated smoking during the period	4.20%	4.72%
RIP (Affordability)	19.87 (SD=6.53)	7.63 (SD =0.86)
Offered free cigarettes	12.44%	8.13%
Sex (male =1) sample	53.62%	48.73%
At least one parent smoke	11.78%	-
Family/class discuss about tobacco	72.50%	51.47%
At least a friend smoke	15.94%	-
Exposed to tobacco adverts	40.46%	56.03%
Age (Years)	14.15 (SD=1.7)	14.10 (SD =1.03)
Heard anti-smoking campaigns	74.64%	57.26%
Age at initiation (Years)	11.95 (SD = 2.9)	11.26 (SD=2.41)
Percentage of initiators before age 16	77%	94%
Percentage of initiators who are males	67%	59%
Awareness of smoke free policies	-	78.24%

3.2. Regression Results

Results from the logit regressions for the unmatched and matched samples are reported in Tables 2 (GYTS 2000 -2009) and 3 (GYTS 2017). The results show a statistically significant and negative relationship between RIP and smoking initiation. For instance, the results for the full unmatched sample (model 1, Table 2) show that OR on RIP is 0.98. This implies that a unit increase in affordability is associated with 0.98 odds of initiating smoking (OR = 0.98, $p<0.05$). Thus, an adolescent who is subjected to a unit increase in RIP has 2% (i.e., 1 – 0.98) × 100 = 2%)) lower probability of initiating smoking than his/her counterpart who is not subjected to the same increase. Note that these results are not elasticities. Females have a

For peer review only - http://bmjopen.bmj.com/site/about/guidelines.xhtml

40.139% (i.e., 1 – 0.599613) × 100 = 40.139%)) lower probability of initiating smoking (OR = 0.599613, p<0.01) compared to their male counterpartss in the unmatched sample.

Table 2: Effect of RIP on smoking initiation among adolescents (GYTS 2000 -2009)

	(Unmatched Sample)	Matched Sample
	Model 1	Model 2
VARIABLES	Odds Ratio	Odds Ratio
RIP	0.980**	0.971***
	(0.008)	(0.009)
Sex (Female =1)	0.613***	0.744**
	(0.074)	(0.091)
Offered free cigarette	1.444**	0.717**
	(0.211)	(0.101)
At least one parent smokes	2.097***	0.872
	(0.276)	(0.107)
Family/class discussion on tobacco	1.001	1.422***
	(0.133)	(0.190)
At least one friend smokes	4.049***	1.082
	(0.513)	(0.126)
Exposed to tobacco adverts	1.136	1.086
	(0.139)	(0.132)
Age	0.393	1.075
	(0.197)	(0.609)
Antismoking message	1.352	1.486**
	(0.218)	(0.238)
Constant	0.885	0.113
	(3.169)	(0.460)
Observations (person-period)	106,673	7,469
Number of people	15,201	1,189
Pseudo R-squared	0.0822	0.0304
Chi-squared	437.3***	75.41***

Standard errors in parentheses

*** p<0.01, ** p<0.05, * p<0.1

	Unmatched	Matched
VARIABLES	Odds ratio	Odds ratio
AAffordability (RIP)	0.981**	0.974***
	(0.009)	(0.009)
Offered free cigarette (Ref=no)	1.491***	0.517***
	(0.216)	(0.071)
Sex (Rref = male)	0.599***	0.615***
	(0.072)	(0.076)

For peer review only - http://bmjopen.bmj.com/site/about/guidelines.xhtml

At least one parent smokes (Rref =no)	2.131***	0.862
	(0.280)	(0.104)
Family/class discussion (Ref = no)	1.001	1.711***
	(0.133)	(0.230)
At least one friend smokes (Rref=no)	4.109***	1.094
	(0.520)	(0.126)
Exposure to adverts (Rref=no)	1.155	1.027
	(0.140)	(0.121)
Age	1.150***	0.991
	(0.042)	(0.031)
Heard of anti-smoking message/campaign (Ref=no)	1.342*	2.048***
	(0.217)	(0.321)
Survey cycle (ref=2000)		
2006	0.958	0.880
	(0.146)	(0.138)
2009	1.108	1.003
	(0.171)	(0.159)
Log (time)	1.110	1.393***
	(0.106)	(0.146)
Constant	0.000***	0.048***
	(0.000)	(0.024)
Observations	106,673	10,078
Number of people	15,201	1,611
Ever-smokers	611	611
Pseudo R-squared	0.0815	0.0448
Cchi2	439.2***	91.84***

Robust standard errors in parentheses
*** p<0.01, ** p<0.05, * p<0.1

For peer review only

Table 3: **Effect of RIP on smoking initiation among adolescents** (GYTS 2017)

	Unmatched	Matched
VARIABLES	Odds ratio	Odds ratio
Affordability (RIP)	0.821**	0.804***
	(0.066)	(0.065)
Sex (Ref=male)	0.659**	0.902
	(0.120)	(0.177)

Offered free cigarettes ~~free~~ (Ref= no)	3.403***	0.978
	(0.726)	(0.221)
Heard of anti-smoking message (Ref=no)	1.165	1.009
	(0.213)	(0.192)
Exposed to tobacco adverts (R~~r~~ef= no)	3.030***	1.893***
	(0.622)	(0.421)
Smoke free policies awareness (Ref=no)	1.250	1.160
	(0.329)	(0.332)
Age	1.847***	1.793***
	(0.294)	(0.279)
Class discussion on tobacco harms (Ref=no)	0.795	1.278
	(0.138)	(0.241)
Log(time)	0.104***	0.119***
	(0.067)	(0.076)
Constant	0.000***	0.002***
	(0.000)	(0.002)
Observations	37,654	4,850
Number of people	5,301	747
Ever-smokers	231	206
Pseudo R-squared	0.0599	0.0292
C~~c~~hi2	158***	38.72***

Robust standard errors in parentheses
*** p<0.01, ** p<0.05, * p<0.1

Similarly, in Table 3, in the unmatched sample, an adolescent faced with a unit increase in RIP has about 18% (i.e., 1 – 0.821) × 100 = 17.9%)) lower probability of starting smoking than his/her counterpart who is not subjected to the same increase.

Other significant factors that influence smoking initiation in our two samples include whether the adolescent's parents (OR = 2.131~~097~~, p<0.01) or friends (OR = 4.1049, p<0.01) smoke. In addition, adolescents who are offered free cigarettes have a high probability of initiating smoking (OR = 1.49144, p<0.01~~5~~) compared to those who receive no such offer for the 2000 -2009 wave (Table 2). In the 2017 wave, the odds of adolescents starting smoking when given cigarettes freely is 3.403 (p<0.01) ~~and statistically significant at conventional levels~~ (Table 3).~~.~~

For peer review only - http://bmjopen.bmj.com/site/about/guidelines.xhtml

In the matched sample (model 2, Table 2), 611 ever-smokers were matched to their two nearest neighbors (1000578 never-smokers) which produced a sample of 1,611189 adolescents with similar characteristics. An adolescent subjected to a unit increase in RIP has a 0.97 times lower chance of initiating smoking (OR = 0.9741, p<0.01) compared to those not exposed to the same increase in RIP. Similarly in the matched sample of the GYTS 2017 (Table 3), a unit increase in RIP is associated with 0.80 times lower odds of smoking initiation (OR = 0.804, p<0.01).

Getting free cigarettes (OR = 0.5717, p<0.015), family/class discussion on tobacco (OR = 1.711422, p<0.01), and hearing antismoking messages (OR = 2.0481.486, p<0.015) are all found to be statistically significant in influencing smoking initiation in the matched sample (Table 2). However, the odds for these variables are contrary to *a priori* expectations. Similarly, in the matched sample (Table 2), the likelihood of initiating smoking among females is lower. The results imply that females have about a 3926% lower probability of initiating smoking (OR = 0.615744, p<0.015) than males.

3.3. Affordability Elasticities

In the unmatched sample, the estimated affordability elasticity is -0.3724 [CI: -0.701692 to -0.04257] for the 2000-2009 sample and -1.247 [-2.248 to -0.0.246] for the 2017 sample., respectively. These elasticities are and it is statistically significant at the 5% level. By sex, the affordability elasticity is -0.137238 for males and -0.888685 for females for the 2000-2009 sample, but only that of females is statistically significant (p<0.01).at the 5% level). The elasticities are higher for 2017 GYTS (-0.938 for males and -1.610 for females), although only that of males is statistically significant. The results are presented in Table 34.

Table 34: Affordability Elasticity estimates (unmatched sample)

VARIABLE	Both sexes	Males	Female
RIP	-0.374**	-0.238	-0.685**

For peer review only - http://bmjopen.bmj.com/site/about/guidelines.xhtml

	(0.162)	(0.199)	(0.274)
95% CI	-0.692 to -0.057	-0.628 to 0.153	-1.221 to -0.149
Observations	106,673	55,396	51,277

Standard errors in parentheses. *** p<0.01, ** p<0.05, * p<0.1

	Both sexes		Male		Female	
Panel A: 2000 -2009						
	Percentage changes (d(lny)/d(lnx))					
VARIABLES	Unmatched	Matched	Unmatched	Matched	Unmatched	Matched
Affordability	-0.372**	-0.490***	-0.137	-0.326	-0.888***	-0.928***
	(0.168)	(0.168)	(0.219)	(0.216)	(0.253)	(0.258)
95% CI	-0.701 to -0.042	-0.818 to -0.161	-0.567 to 0.292	-0.749 to 0.097	-1.384 to -0.392	-1.434 to -0.422
Observations	106,673	10,078	55,396	5,648	51,277	4,430
Panel B: 2017 GYTS						
Affordability	-1.247**	-1.349***	-0.938**	-1.045**	-1.610*	1.518**
	(0.511)	(0.500)	(0.474)	(0.484)	(0.866)	(0.778)
95% CI	-2.248 to -0.246	-2.328 to -0.369	-1.867 to -0.008	-1.993 to -0.096	-3.307 to -0.087	-3.043 to -0.007
Observations	37,654	4,850	18,084	2,807	19,570	2,043

Standard errors in parentheses
*** p<0.01, ** p<0.05, * p<0.1

In the matched sample for the 2000-2009 GYTS, Table 4 presents the affordability elasticity for the matched sample. tThe overall elasticity is -0.490547 [CI: -0.8168 to -0.12261] for both sexes (Table 434), which is similar to that of the unmatched sample in Table 4. For males, athe effect of changes in percentage increase in RIP iswas statistically insignificant. reduces the likelihood of initiating smoking by 0.544% and is statistically significant at 1%. Among females, a percentage increase in RIP is associated with a 0.928551% lower probability of smoking initiation. The elasticities for both males and females in the 2017 GYTS were negative, statistically significant -and more than unity.

Table 4: Affordability Elasticity estimates after matching.

Variables	Both sexes	Males	Females
RIP	-0.547***	-0.544***	-0.551***
	(0.163)	(0.163)	(0.164)

95% CI	-0.868 to -0.226	-0.864 to -0.224	-0.874 to -0.229
Observations	7469	4522	2947

Standard errors in parentheses. *** p<0.01, ** p<0.05, * p<0.1

The mean and median standardized difference for the covariates used in matching show that the matching satisfies the balancing test (results not reported). The mean and median standardized difference is 2.3%, which is below the normal 10% threshold. Therefore, the balancing property is satisfied.

5. DISCUSSION AND CONCLUSION

In this study, increasing the RIP of cigarettes is significantly associated with a lower probability of initiating smoking. This finding is consistent with international literature, including the few existing studies in SSA that have reported that making cigarettes less affordable lowers the likelihood of smoking initiation among young people.[17 18 26] In addition, the results from the unmatched 2000-2009 sample suggest that males are not responsive to changes in RIP whilst females are. Nevertheless, in the matched sample analysis, especially using the 2017 GYTS, both males and femalesboys and girls arewere responsive to changes in RIP, at least for the 2017 GYTS... Indeed, the issue of affordability becomes more important given that Ghana's per capita income has been growing at an average of 4.4% annually in the last decade.[30]

Parental smoking increased the odds of smoking initiating. This points to the parental influence on the lifestyle of adolescents. Adolescents whose parents smoke may perceive smoking as acceptable behavior. Previous studies have reported similar findings.[10 27 31 53] The odds of smoking initiation is higher for those whose friends' smoke. This points to the

For peer review only - http://bmjopen.bmj.com/site/about/guidelines.xhtml

influence of peers, and is consistent, for example, with Kwok-Kei Mak, et al. [31]. Those who were offered free cigarettes by sales representatives were more likely to initiate smoking. All forms of tobacco promotion and advertising were banned in Ghana in 1982. However, the tobacco industry seems to be breaking these laws, because 12.44% of youth reported being offered a cigarette for free. This observation, together with our results, suggest the need to strengthen the enforcement of the existing ban on all forms of tobacco advertising and promotion in Ghana.

This study has several limitations. The GYTS is a self-reporting survey, which means the responses are prone to recall bias even in cases where the adolescents are required to answer questions about events that occurred in the past 30 days. For instance, students may not recall the exact age at which they tried their first puff. There is also a social desirability bias when self-reporting behaviors such as smoking, especially among females. The weakness of our measure of smoking initiation is that it may not predict regular smoking behavior well.[41 42] In addition, there are other important factors affecting smoking uptake among the youth that are not included in this study. For instance, changing community norms regarding smoking, the enforcement of laws regarding the sale of cigarettes to minors, and changes in the social image of smoking are key factors that may influence smoking participation,[54 55] but are not included in the models estimated.

In conclusion, making cigarettes less affordable is associated with a lower probability of smoking initiation among adolescents in Ghana. This supports the use of price measures, through higher excise taxes, as effective strategies to decrease smoking initiation among adolescents in Ghana. Since ~~With rising~~ incomes are rising at the average of 4.4% annually[30], tobacco taxes need to be adjusted regularly to ensure that cigarettes or other tobacco products become less affordable over time in order to discourage young people from initiating smoking and to encourage smokers to quit.

For peer review only - http://bmjopen.bmj.com/site/about/guidelines.xhtml

Author Contributions: Conceptualization, M.K.B.; methodology, M.K.B; software, M.K.B.; validation, M.K.B.., M.I., E.N.T., N.D.M., and H.R.; formal analysis, M.K.B.; investigation, M.K.B., M.I., E.N.T., N.D.M., and H.R.; data curation, M.K.B; writing—original draft preparation, M.K.B., M.I., E.N.T., N.D.M., and H.R.; writing—review and editing, M.K.B.~~.~~, M.I., E.N.T., N.D.M., H.R.; project administration, M.K.B.; supervision, H.R.; funding acquisition, H.R. All authors have read and approved the manuscript.

Funding: Funding for this study was provided by Cancer Research UK (Grant number: C62640/A24723) and International Development Research Centre (Grant Number: 108820-001), with~~.~~ additional support ~~comes~~ from the SAMRC/Wits Centre for Health Economics and Decision Science – PRICELESS SA (grant number 23108). Funding for ENT and NDM is acknowledged from the Tobacco Control Capacity Programme (Grant Number: MR/P027946/2) supported by UK Research and Innovation (UKRI) with funding from the Global Challenges Research Fund (GCRF). The funders had no role in study design, data collection and analysis, or preparation of the manuscript.

For peer review only

Conflicts of Interest: The authors declare that they have no conflict of interest.

Research Ethics Approval: Human Participants

This study does not involve human participants.

Research Ethics Approval: Animals

This study does not involve animal subjects.

Patient consent for publication: No patient involved.

Data Availability statement: The publicly available data can be accessed: https://nccd.cdc.gov/GTSSDataSurveyResources/Ancillary/DataReports.aspx?CAID=2. The relative income price data are available from the authors on reasonable request.

REFERENCES

1. Doll R, Peto R, Boreham J, et al. Mortality in relation to smoking: 50 years' observations on male British doctors. *Bmj* 2004;328(7455):1519.
2. Winkler V, Mangolo NJ, Becher H. Lung cancer in South Africa: a forecast to 2025 based on smoking prevalence data. *BMJ open* 2015;5(3):e006993.
3. World Health Organization. Tobacco: Key Facts. 2020 [updated 27 May 2020. Available from: https://www.who.int/news-room/fact-sheets/detail/tobacco accessed 22 November 2020.
4. Goodchild M, Nargis N, d'Espaignet ET. Global economic cost of smoking-attributable diseases. *Tobacco Control* 2018;27(1):58-64.
5. Boachie MK, Rossouw L, Ross H. The Economic Cost of Smoking in South Africa, 2016. *Nicotine Tob Res* 2021;23(2):286 - 93. doi: 10.1093/ntr/ntaa162
6. USDHHS. Preventing tobacco use among youth and young adults: A report of the surgeon general 2012 [Available from: http://www.surgeongeneral.gov/library/reports/preventing-youth-tobacco-use/full-report.pdf (accessed: Jan 3, 2019).
7. World Health Organization. WHO global report on trends in prevalence of tobacco use 2000-2025. 3rd ed. Geneva: World Health Organization, 2019.
8. CDC, WHO, MOH. GLOBAL YOUTH TOBACCO SURVEY: Factsheet, Ghana 2017, 2018.
9. World Health Organization. WHO global report on trends in prevalence of tobacco smoking 2015. Geneva: World Health Organization, 2015.
10. Vellios N, Van Walbeek C. Determinants of regular smoking onset in South Africa using duration analysis. *BMJ Open* 2016;6(7):e011076.
11. IARC. Effectiveness of tax and price policies for tobacco control. In: Cancer. IAfRo, ed. USA: International Agency for Research on Cancer, 2011.
12. Boachie MK, Ross H. Determinants of smoking intensity in South Africa: evidence from township communities. *Preventive Medicine Reports* 2020;19:101099.

For peer review only - http://bmjopen.bmj.com/site/about/guidelines.xhtml

13. Van Walbeek C. A simulation model to predict the fiscal and public health impact of a change in cigarette excise taxes. *Tobacco control* 2010;19(1):31-36.

14. Yurekli A, de Beye J. Design and administer tobacco taxes. *World Bank economics of tobacco toolkit* 2001(4)

15. Van Walbeek C. Raising Additional Government Revenues in Ghana by Raising the Excise Tax on Tobacco and Alcohol Washington, D.C.: WBG Global Tobacco Control Program, World Bank Group; 2014 [Available from: http://documents.worldbank.org/curated/en/133561561707742558/pdf/Raising-Additional-Government-Revenues-in-Ghana-by-Raising-the-Excise-Tax-on-Tobacco-and-Alcohol.pdf accessed 15 July 2019.

16. He Y, Shang C, Chaloupka FJ. The association between cigarette affordability and consumption: An update. *PLOS ONE* 2018;13(12):e0200665. doi: 10.1371/journal.pone.0200665

17. Nargis N, Stoklosa M, Shang C, et al. Price, Income, and Affordability as the Determinants of Tobacco Consumption: A Practitioner's Guide to Tobacco Taxation. *Nicotine and Tobacco Research* 2021;23(1):40-47.

18. Blecher E, Van Walbeek C. An international analysis of cigarette affordability. *Tobacco Control* 2004;13(4):339-46.

19. Ding A. Youth are more sensitive to price changes in cigarettes than adults. *The Yale journal of biology and medicine* 2003;76(3):115.

20. Ho L-M, Schafferer C, Lee J-M, et al. The effect of cigarette price increases on cigarette consumption, tax revenue, and smoking-related death in Africa from 1999 to 2013. *International journal of public health* 2017;62(8):899-909.

21. Chelwa G, van Walbeek C. Does cigarette demand respond to price increases in Uganda? Price elasticity estimates using the Uganda National Panel Survey and Deaton's method. *BMJ open* 2019;9(3):e026150. doi: 10.1136/bmjopen-2018-026150 [published Online First: 2019/03/23]

For peer review only - http://bmjopen.bmj.com/site/about/guidelines.xhtml

22. Stoklosa M, Goma F, Nargis N, et al. Price, tax and tobacco product substitution in Zambia: findings from the ITC Zambia Surveys. *Tob Control* 2019;28(Suppl 1):s45-s52. doi: 10.1136/tobaccocontrol-2017-054037 [published Online First: 2018/03/27]

23. Mukong AK, Tingum EN. The demand for cigarettes: New evidence from South Africa. *Development Southern Africa* 2020;37(1):40-54.

24. Tingum EN, Mukong AK, Mdege N. The effects of price and non-price policies on cigarette consumption in South Africa. *Tob Induc Dis* 2020;18(July) doi: 10.18332/tid/123424

25. Kidane A, Mduma J, Naho A, et al. The Demand for Cigarettes in Tanzania and Implications for Tobacco Taxation Policy. *Advances in economics and business* 2015;3(10):428.

26. Kostova D. A (nearly) global look at the dynamics of youth smoking initiation and cessation: the role of cigarette prices. *Applied Economics* 2013;45(28):3943-51.

27. Asare S, Stoklosa M, Drope J, et al. Effects of Prices on Youth Cigarette Smoking and Tobacco Use Initiation in Ghana and Nigeria. *International Journal of Environmental Research and Public Health* 2019;16(17):3114.

28. Ministry of Health. Needs assessment for the Implementation of WHO FCTC in Ghana. Accra: Ministry of Health,, 2010.

29. Gov't. of Ghana. Customs and Excise (Duties and Other Taxes) Amendment Act 739. , 2007.

30. World Bank. World Development Indicators.: World Bank; 2019 [Available from: https://databank.worldbank.org/source/world-development-indicators. accessed 01 Jan 2020.

31. Mak K-K, Ho S-Y, Day JR. Smoking of parents and best friend—independent and combined effects on adolescent smoking and intention to initiate and quit smoking. *Nicotine Tob Res* 2012;14(9):1057-64.

32. Guindon GE, Paraje GR, Chávez R. Prices, inflation, and smoking onset: the case of Argentina. *Economic Inquiry* 2018;56(1):424-45.

33. Dauchy E, Ross H. The Effect of Price and Tax Policies on the Decision to Smoke among Men in Kenya. *Addiction* 2019;114(7):1249-63.

For peer review only - http://bmjopen.bmj.com/site/about/guidelines.xhtml

34. Li M. Using the propensity score method to estimate causal effects: A review and practical guide. *Organizational Research Methods* 2013;16(2):188-226.

35. Warren CW. Tobacco use among youth: a cross country comparison. *Tobacco Control* 2002;11(3):252-70.

36. Gov't. of Ghana. Public Health Act, 2012 Act 851. Ghana 2012.

37. Wellington E. Ghana Global Youth Tobacco Survey (GYTS), Factsheet. 2006 [Available from: http://www.afro.who.int/en/downloads/docdownload/1887-ghana-gyts-2006-factsheet.html (Retrieved Jan 28, 2019).

38. Cadmus EO, Ayo-Yusuf OA. The effect of smokeless tobacco use and exposure to cigarette promotions on smoking intention among youths in Ghana. *Cogent Medicine* 2018;5(1):1531459.

39. CDC and WHO. Ghana global youth tobacco survey. Atlanta, United States: Centers for Disease Control and Prevention (CDC). 2006.

40. Freedman KS, Nelson NM, Feldman LL. Smoking initiation among young adults in the United States and Canada, 1998-2010: a systematic review. *Preventing chronic disease* 2012;9

41. Azagba S, Baskerville NB, Minaker L. A comparison of adolescent smoking initiation measures on predicting future smoking behavior. *Preventive medicine reports* 2015;2:174-77.

42. Reidpath DD, Davey TM, Kadirvelu A, et al. Does one cigarette make an adolescent smoker, and is it influenced by age and age of smoking initiation? Evidence of association from the US Youth Risk Behavior Surveillance System (2011). *Preventive medicine* 2014;59:37-41.

43. Centers for Disease Control Prevention. Selected cigarette smoking initiation and quitting behaviors among high school students--United States, 1997. *MMWR Morbidity and mortality weekly report* 1998;47(19):386.

44. Blecher E. Targeting the affordability of cigarettes: a new benchmark for taxation policy in low-income and-middle-income countries. *Tobacco control* 2010;19(4):325-30.

For peer review only - http://bmjopen.bmj.com/site/about/guidelines.xhtml

45. Nargis N, Stoklosa M, Drope J, et al. Trend in the affordability of tobacco products in Bangladesh: findings from the ITC Bangladesh Surveys. *Tobacco control* 2019;28(Suppl 1):s20-s30.

46. World Health Organization. WHO report on the global tobacco epidemic 2019: Offer help to quit tobacco use. 2019

47. Philip Morris International. World summary of cigarette retail prices, tax burdens and tax incidence.: Philips Moris Records 1996 [Available from: https://www.industrydocuments.ucsf.edu/docs/#id=glhf0066.

48. Ghana Gto. Report card on the WHO Framework Convention on Tobacco Control., 2009.

49. Ghana Statistical Service. Consumer Price Index (CPI): Time Series In: Service GS, ed. Accra, 2012.

50. Austin PC, Jembere N, Chiu M. Propensity score matching and complex surveys. *Statistical methods in medical research* 2018;27(4):1240-57.

51. DuGoff EH, Schuler M, Stuart EA. Generalizing observational study results: applying propensity score methods to complex surveys. *Health services research* 2014;49(1):284-303.

52. Jenkins SP. Survival Analysis with Stata. [Available from: https://www.iser.essex.ac.uk/resources/survival-analysis-with-stata accessed 01 January 2020.

53. Gilman SE, Rende R, Boergers J, et al. Parental smoking and adolescent smoking initiation: an intergenerational perspective on tobacco control. *Pediatrics* 2009;123(2):e274-e81.

54. Gilpin EA, Pierce JP. Trends in adolescent smoking initiation in the United States: is tobacco marketing an influence? *Tobacco Control* 1997;6(2):122-27.

55. Cawley J, Markowitz S, Tauras J. Obesity, cigarette prices, youth access laws and adolescent smoking initiation. *Eastern Economic Journal* 2006;32(1):149-70.

For peer review only - http://bmjopen.bmj.com/site/about/guidelines.xhtml

BMJ Open

Effect of relative income price on smoking initiation among adolescents in Ghana: evidence from pseudo-longitudinal data

Journal:	*BMJ Open*
Manuscript ID	bmjopen-2021-054367.R2
Article Type:	Original research
Date Submitted by the Author:	22-Jan-2022
Complete List of Authors:	Boachie, Micheal Kofi; University of the Witwatersrand, Centre Health Economics and Decision Science - PRICELESS SA<br>Immurana, Mustapha; University of Health and Allied Sciences, Institute of Health Research<br>Tingum, Ernest; University of Cape Town, Economics<br>Mdege, Noreen; University of York, Health Sciences<br>Ross, Hana; University of Cape Town, School of Economics
**Primary Subject Heading**:	Global health
Secondary Subject Heading:	Health economics, Global health, Public health, Smoking and tobacco
Keywords:	HEALTH ECONOMICS, PUBLIC HEALTH, SOCIAL MEDICINE

SCHOLARONE™
Manuscripts

I, the Submitting Author has the right to grant and does grant on behalf of all authors of the Work (as defined in the below author licence), an exclusive licence and/or a non-exclusive licence for contributions from authors who are: i) UK Crown employees; ii) where BMJ has agreed a CC-BY licence shall apply, and/or iii) in accordance with the terms applicable for US Federal Government officers or employees acting as part of their official duties; on a worldwide, perpetual, irrevocable, royalty-free basis to BMJ Publishing Group Ltd ("BMJ") its licensees and where the relevant Journal is co-owned by BMJ to the co-owners of the Journal, to publish the Work in this journal and any other BMJ products and to exploit all rights, as set out in our licence.

The Submitting Author accepts and understands that any supply made under these terms is made by BMJ to the Submitting Author unless you are acting as an employee on behalf of your employer or a postgraduate student of an affiliated institution which is paying any applicable article publishing charge ("APC") for Open Access articles. Where the Submitting Author wishes to make the Work available on an Open Access basis (and intends to pay the relevant APC), the terms of reuse of such Open Access shall be governed by a Creative Commons licence – details of these licences and which Creative Commons licence will apply to this Work are set out in our licence referred to above.

Other than as permitted in any relevant BMJ Author's Self Archiving Policies, I confirm this Work has not been accepted for publication elsewhere, is not being considered for publication elsewhere and does not duplicate material already published. I confirm all authors consent to publication of this Work and authorise the granting of this licence.

For peer review only

Effect of relative income price on smoking initiation among adolescents in Ghana: evidence from pseudo-longitudinal data

Micheal Kofi Boachie[1, 2], Mustapha Immurana[3], Ernest N. Tingum[2, 4], Noreen Dadirai Mdege[5], Hana Ross[2]**

[1] SAMRC/Wits Centre for Health Economics and Decision Science – PRICELESS SA, School of Public Health, University of the Witwatersrand, Johannesburg 2193, South Africa; mkboachie@gmail.com

[2] Research Unit on the Economics of Excisable Products (REEP), School of Economics, University of Cape Town, Cape Town, South Africa; hana.ross@uct.ac.za

[3] Institute of Health Research, University of Health and Allied Sciences, Ho, Ghana; mimmurna@uhas.edu.gh; mustaphaimmurana@gmail.com

[4] Department of Economics, University of Namibia, Windhoek, Namibia; ngehernest@gmail.com

[5] Department of Health Sciences, University of York, Heslington, United Kingdom; noreen.mdege@york.ac.uk

** Correspondence: mkboachie@gmail.com; Tel.: +27 845 644 650.

Word count:

Abstract: 290

Introduction: 714

Materials and Methods: 1507

Results: 1330

Discussion and Conclusion: 508

Total word count: 4349

Number of References: 54

For peer review only

Abstract:

Objective: Many smokers initiate smoking during adolescence. Making tobacco products less affordable is one of the best ways to control tobacco use. Studies on the effect of relative income price (RIP (i.e., affordability)) of cigarettes on smoking initiation are scarce in low- and middle-income countries, especially in Sub-Saharan Africa where data are limited. The goal of this study is to examine the effect of cigarette RIP on adolescent smoking initiation in Ghana.

Setting: The study uses a pseudo-longitudinal dataset constructed from the Global Youth Tobacco Surveys (GYTS (2000-2009 and 2017)) and RIP for the most sold cigarette brand in Ghana.

Participants: The GYTS is a national survey on adolescents.

Primary and secondary outcome: Effect of RIP on adolescent smoking initiation in Ghana.

Results:

Using the GYTS 2000-2009 data, we find that the probability of smoking initiation falls significantly in response to a higher RIP, with an elasticity of -0.372 (95% CI: -0.701 to -0.042) for the unmatched sample and -0.490 (95% CI: -0.818 to -0.161) for the matched sample. The RIP elasticity for females [(-0.888) (95% CI: -1.384 to -0.392) and (-0.928) (95% CI: -1.434 to -0.422)] is statistically significant at 1% in both the unmatched and the matched samples, respectively, while the RIP elasticity for males is statistically insignificant in the 2000 - 2009 surveys. Analysis of the 2017 GYTS shows a similar outcome: a negative relationship between RIP and smoking initiation, and the results are statistically significant for both male and female, and for both matched and unmatched samples.

For peer review only

Conclusion: The affordability (RIP) of cigarettes is negatively related to the probability of smoking initiation among adolescents in Ghana. Raising tobacco taxes in line with income growth would make cigarettes less affordable and dissuade adolescents from initiating smoking.

Keywords: prices; taxes; smoking initiation; adolescents; relative income price

For peer review only

Strengths and Limitations of the study

- This is the first study to explore the impact of Relative Income Price (i.e., affordability index) of cigarettes on youth smoking initiation in Sub-Saharan Africa.
- Our analysis controls for variables that are known, empirically or theoretically, to be associated with smoking initiation, and the relationship is tested using a pseudo-longitudinal dataset of 17 years.
- We also examine potential sex differences in the effect of affordability on cigarette smoking initiation: this is key to the implementation of tobacco control policies that confer adequate protection across both genders.
- Since GYTS data are available in many low- and middle-income countries (LMIC), our study provide a template on how to do analyses elsewhere in order to enhance our understating of the impact of cigarette affordability on smoking uptake in LMIC.

For peer review only - http://bmjopen.bmj.com/site/about/guidelines.xhtml

- The results are subject to self-reporting and recall biases as well as omitted variable bias due to lack of data on other factors affecting smoking uptake.

For peer review only

For peer review only - http://bmjopen.bmj.com/site/about/guidelines.xhtml

1. INTRODUCTION

Tobacco use is one of the major risk factors for many non-communicable diseases such as lung cancer and ischemic heart disease,[1 2] and it accounts for over eight million deaths annually worldwide.[3] Tobacco use also imposes huge financial burdens on households and governments.[4 5] The majority (80-90%) of the one billion adult smokers globally began the habit during their adolescence.[6] The current smoking prevalence in Ghana is still low relative to other African countries (3.2% among adults aged 15 years and older,[7] and 6.4% among students aged 13-15 years [8]). However, the number of smokers is predicted to increase from 1.3 million to 1.7 million (i.e., by about 30%) between 2020 and 2025.[9] The expected increase in the number of smokers will be partly driven by initiation among adolescents, therefore lowering initiation is key to slowing down the tobacco epidemic in Ghana.

Empirical evidence shows that tobacco consumption (initiation and intensity) is significantly inversely related to price.[10-12] In addition to being the most cost-effective measure to reduce tobacco use, increasing the taxes of tobacco products generates revenue for governments.[13-15] Nevertheless, economic factors such as income growth can negatively affect the response of tobacco consumption to tax/price changes.[16 17] Increasing tobacco prices can be more effective in reducing tobacco consumption if it reduces affordability.[18] Affordability (relative income price (RIP)) elasticity which measures the sensitivity of consumers to real changes in both price and income, can therefore be a useful parameter to explain and predict the sensitivity of consumers to tobacco tax and price policies even in the presence of income growth.[17] This is particularly important for tobacco control measures aimed at adolescents because they are more price sensitive than adults,[12 19] for instance in Ghana where 71.3% of current cigarette smokers aged 13-15 buy their own cigarettes.[8]

For peer review only - http://bmjopen.bmj.com/site/about/guidelines.xhtml

Some Sub-Saharan African (SSA) studies, albeit few, have demonstrated that increasing higher cigarette prices reduces smoking prevalence and intensity of use.[12 20-25] However, there is a scarcity of studies on the relationship between cigarette prices and smoking initiation in the SSA context. One study, using data from 48 countries, including four from SSA (Kenya, Nigeria, Senegal, and South Africa), concluded that higher cigarette prices reduce smoking initiation in early youth, with girls being more responsive than boys.[26] However, findings from the SSA countries were not reported separately from the overall study findings. A study in South Africa reported a significant reduction in regular smoking initiation among males due to higher cigarette prices, but not among females [10]. Another study in Nigeria and Ghana concluded that increasing cigarette prices resulted in a reduction in both 30-day cigarette smoking and cigarette smoking onset in both countries.[27]

Ghana has implemented a number of tobacco tax changes over the last 20 years. For example, it implemented a specific excise tax in 2008 and subsequently switched to an ad valorem tax structure in 2010.[28 29] At the same time, per capita income in Ghana has been growing at an average rate of 4.4% annually in the last decade.[30] These changes have implications for the retail prices of tobacco products (e.g., cigarettes), and the affordability of cigarettes or other tobacco products. However, to our knowledge, no study has analyzed the impact of cigarette affordability on smoking initiation in Ghana.

We address this critical evidence gap by examining the effect of cigarette affordability on smoking initiation among adolescents in Ghana. We hypothesize that making cigarettes less affordable reduces the likelihood of smoking initiation among young people, and we make use of the Global Youth Tobacco Survey (GYTS) data and other datasets to test that hypothesis. The control variables used are sex, age, parents', and friends' smoking status, being offered a cigarette for free, family/class discussion about tobacco, exposure to antismoking messages, and exposure to tobacco advertisements. These controls are based on

For peer review only - http://bmjopen.bmj.com/site/about/guidelines.xhtml

variables that are known, empirically or theoretically, to be associated with smoking initiation.[27 31]

Our analysis addresses the potential endogeneity of price, if any, as a driver of cigarette demand through i) using aggregate level prices and not self-reported prices[32] and ii) the use of propensity score matching techniques.[33 34] We also examine potential sex differences in the effect of affordability on cigarette smoking initiation. An understanding of these dynamics is key to the implementation of context-specific tobacco and non-communicable disease control policies in Ghana.

2. MATERIALS AND METHODS

2.1. Data and variables

We make use of three waves (2000, 2006 and 2009) of the GYTS and RIP (affordability) data (1991–2009) to analyze the effect of affordability on smoking initiation among adolescents in Ghana. The World Health Organization (WHO) defines adolescents as young people between the ages of 10 and 19 years. The GYTS questionnaire specifies ages from 11 years or younger to 17 years or higher for current age (i.e., age at the time of survey). It also captures age at first puff, which ranges from 7 years or younger to 16 years or older. For the purposes of this study, we classify respondents as adolescents, youth or young people. The terms are used interchangeably in the study. The GYTS is a school-based survey developed to enhance the capacity of countries to monitor tobacco use among the youth, as well as implement and evaluate tobacco control and prevention programs.[35] These data provide representative trends of tobacco use among adolescents. The GYTS is a cross-sectional survey and does not follow individuals over time, but provides data on smoking patterns among adolescents. In countries where it is conducted at regular intervals, it allows the monitoring of trends over time. We are aware of the 2017 GYTS for Ghana, but we do not include it in the analysis of the pooled

For peer review only - http://bmjopen.bmj.com/site/about/guidelines.xhtml

2000 – 2009 surveys due to inconsistencies in the questions asked, compared to those in previous GYTS surveys. There is no other survey on adolescents in Ghana with comparable measures except the ones outlined. We analyze the 2017 GYTS separately while linking it with RIP data for 2008 – 2017 based on age-at-risk criteria[27 32 36] (as done for the 2000 – 2009 surveys).

Although the GYTS data contain adolescents whose first puff was at age 7 or younger, we assume that a student is at risk at age 8 because that is the age at which the child is relatively developed and is able to start out-of-home interaction with peers.[27 32 36] Students who started smoking before reaching age 8 and those below age 8 are therefore excluded from the pooled sample and not followed. In line with previous studies, a student exits the sample once smoking is initiated.[10 27 32 33]

In Ghana, there was no law prohibiting the sale of cigarettes to minors until 2012 when restrictions on sale to persons below age 18 years were introduced.[37] The GYTS sample is drawn using a two-stage cluster-sampling design.[35 38 39] Schools are selected with probability proportional to school enrolment size during the first stage, and then classes within participating schools are selected as a systematic equal probability sample with a random start during the second stage. All students in the selected classes are eligible to participate in the survey.

The Ghana GYTS questionnaire captures information on the use of tobacco products such as cigarettes and shisha. The data also include information on parental and peer smoking habits, perception about tobacco use (e.g., weight gain, health effects, and ease of quitting), money spent on tobacco in the last 30 days before the survey, and second-hand smoking (SHS).[40] Studies vary widely on the way they define or measure smoking initiation.[41-43] For the GYTS, smoking initiation is measured using the definition of a lifetime smoker, i.e., a person who

For peer review only

has ever tried smoking, *even one or two puffs of a cigarette*.[27 43 44] Thus, for our study, smoking initiation (dependent variable) is a dichotomous variable generated from the following GYTS question where students answer Yes/No: *Have you ever tried or experimented with cigarette smoking, even one or two puffs?*.[27]

The main independent variable is the affordability index or the RIP, measured as the percentage of GDP per capita (per capita income) required to buy 100 packs of cigarettes (20 sticks per pack, in total 2,000 sticks).[17 18 45 46] Affordability is a relative measure and is calculated using nominal prices and nominal GDP per capita, or real prices and real GDP per capita. Data on per capita income are obtained from the World Bank's World Development Indicators,[30] and those of average cigarette prices (for the most-sold brand) come from the WHO, relevant publications of the tobacco industry[47] and the Government of Ghana[48]. Years with missing data on prices were interpolated using the formula:

$$P_{t-1} = \frac{P_t}{(1 + Tob.Inflation_t)} \quad \ldots\ldots\ldots\ldots\ldots\ldots (1)$$

where P_{t-1} is the previous year's price of cigarette, P_t is current price of cigarette and $Tob.Inflation_t$ is the current tobacco inflation.[49] We then calculate RIP following methods used by preceding studies, with a lower affordability index (RIP) indicating that cigarettes have become more affordable and a higher value indicating that cigarettes have become less affordable relative to the reference year.[17 18 45 46] The WHO uses the same approach to obtain its affordability index. Other independent variables used are sex, age, parents', and friends' smoking status, whether offered cigarettes for free, family/class discussion about tobacco, exposure to antismoking messages, and exposure to tobacco advertisements. These variables are selected as they have been shown, theoretically or empirically, to be associated with

For peer review only - http://bmjopen.bmj.com/site/about/guidelines.xhtml

smoking initiation.[27 31] Except age and RIP, which are continuously measured, all variables are dichotomous.

2.2. Data analysis

We construct a pseudo-longitudinal dataset based on current age and age at first puff. In doing this, we create a historical dataset starting from age 8 (age-at-risk criteria) and follow the person until s/he initiates smoking. This is done by inferring the year of first puff using the GYTS question: "How old were you when you first tried a cigarette?" and the age at the time of the survey.[27]

STATA routine command, *expand*, is used to expand the person's age at the time of the survey after which an event variable indicating smoking status is created. We then link the RIP (affordability index) data to this pseudo-longitudinal dataset.

Our statistical technique is duration or event history analysis where the timing of transition into initiation is a function of the probability of initiating in period *t* conditional on not having experienced a transition until period *t*, known as the hazard rate.[10] Following previous empirical studies,[10 32 36] we employ the discrete time-hazard model, with logit specification (see equation 2), to study the association between RIP (affordability) and smoking initiation among adolescents.

$$\Pr(Initiation = 1 \mid X') = \beta_0 + \beta_1 RIP + \beta_i X' \quad \ldots (2)$$

where Initiation is defined as first cigarette puff, RIP is the affordability index, X' is a vector of other independent variables affecting smoking initiation among adolescents, and β is a vector of the regression coefficients. The predictors, X', represent age, sex, whether offered free cigarette, parental and peer smoking status, family/class discussion on the dangers of tobacco, exposure to tobacco advertisements, and hearing of antismoking messages, and

For peer review only - http://bmjopen.bmj.com/site/about/guidelines.xhtml

awareness of tobacco control policies introduced in 2012 (for the 2017 GYTS). We report odds ratios (ORs), and the statistical level of significance is set at $p \leq 0.1$. OR <1 implies that when a higher share of income is required to buy 2000 cigarettes (cigarettes are less affordable), the risk of smoking initiation declines, and vice versa. The partial derivative of equation 2 with respect to RIP gives the affordability elasticity.

To check the robustness of the logistic regression estimates, we employ a propensity score matching (PSM) technique to match ever-smokers to never-smokers based on the propensity scores. Our approach to matching follows previous studies.[33 50 51] The propensity scores are obtained by running a logit regression to estimate the probability of being a smoker based on the variables in equation 2, except RIP, and the predicted probabilities are then used to match ever-smokers to never-smokers. Using the neighborhood matching, ever-smokers are matched to their two nearest neighbors. After matching the sample, we re-estimate the logit model to assess the effect of affordability on the probability of initiating smoking, using GYTS weights on the matched sample.[50 51] With the matching approach, we are able to obtain the effect of affordability on the probability of initiating smoking among adolescent smokers and non-smokers who possess similar characteristics based on the propensity scores. This technique addresses issues of endogeneity and concerns relating to the fact that some never-smokers will never choose to smoke or use any form of tobacco irrespective of market conditions.[33 34] Further, we minimize the problem of endogeneity by not using self-reported prices.[32] Data analysis is conducted using STATA version 15. The study benefited immensely from discrete time modelling guidelines and STATA code produced by Professor Stephen Jenkins.[52]

2.3 Interpretation of RIP Elasticity

For peer review only - http://bmjopen.bmj.com/site/about/guidelines.xhtml

Although the RIP is measured in percentages, interpretation of the affordability index follows the same procedure for elasticity interpretation. The elasticity measures the percentage change in probability of initiating smoking following a percentage change in RIP, ceteris paribus. Assuming the current RIP is 6% or 0.06, then a 1% increase in RIP corresponds to the current RIP increasing from 6% to 6.06%. When using a unit change interpretation, a unit change will be RIP moving from 6% to 7% and therefore probabilities will change in absolute units and not percentages. Such distinction is important in understanding the impact of affordability on smoking behavior.

Patient and Public Involvement

No patient involved.

3. RESULTS

3.1. Descriptive statistics

A total of 20,202 adolescents were interviewed across the three GYTS waves (2000 – 2009). 54% of the respondents were males, while 76.47% were aged 15 years or less. In the 2017 GYTS, 5,664 people were interviewed, with about 48% being males. Overall, 12.46% and 8.9% of the respondents in the pooled (2000 – 2009) and 2017 surveys, respectively, had ever smoked.

Given our age-at-risk criteria, 15,861 (2000 – 2009 GYTS) and 5,389 (2017 GYTS) people were eligible for inclusion in our pseudo-longitudinal analysis. For surveys prior to 2017, 4.2% initiated smoking at some point between 1991 and 2009, and about 77% of smoking initiators did so before reaching age 16. Further, 67% of initiators were males. Overall, males represented 53.62% of the eligible respondents. In the 2017 survey, 4.72% of the respondents

For peer review only - http://bmjopen.bmj.com/site/about/guidelines.xhtml

initiated smoking at some point between 2008 and 2017. The characteristics of the samples are presented in Table 1. Due to incomplete information on some of the variables, the number of people used in the regression varies.

Table 1: Descriptive statistics

Variable	2000 – 2009 GYTS	2017 GYTS
	Students, n = 15 861	n=5389
Initiated smoking during the period	4.20%	4.72%
RIP (Affordability)	19.87 (SD=6.53)	7.63 (SD =0.86)
Offered free cigarettes	12.44%	8.13%
Sex (male =1)	53.62%	48.73%
At least one parent smoke	11.78%	-
Family/class discuss about tobacco	72.50%	51.47%
At least a friend smoke	15.94%	-
Exposed to tobacco adverts	40.46%	56.03%
Age (Years)	14.15 (SD=1.7)	14.10 (SD =1.03)
Heard anti-smoking campaigns	74.64%	57.26%
Age at initiation (Years)	11.95 (SD = 2.9)	11.26 (SD=2.41)
Percentage of initiators before age 16	77%	94%
Percentage of initiators who are males	67%	59%
Awareness of smoke free policies	-	78.24%

For peer review only

3.2. Regression Results

Results from the logit regressions for the unmatched and matched samples are reported in Tables 2 (GYTS 2000 – 2009) and 3 (GYTS 2017). The results show a statistically significant and negative relationship between RIP and smoking initiation. For instance, the results for the full unmatched sample (Table 2) show that OR on RIP is 0.98. This implies that a unit increase in affordability is associated with 0.98 odds of initiating smoking (OR = 0.98, $p<0.05$). Thus, an adolescent who is subjected to a unit increase in RIP has 2% (i.e., 1 –

For peer review only - http://bmjopen.bmj.com/site/about/guidelines.xhtml

0.98) × 100 = 2%)) lower probability of initiating smoking than his/her counterpart who is not subjected to the same increase. Note that these results are not elasticities. Females have 40.1% (i.e., 1 – 0.599) × 100 = 40.1%)) lower probability of initiating smoking (OR = 0.599, $p<0.01$) compared to their male counterparts in the unmatched sample.

Table 2: Effect of RIP on smoking initiation among adolescents (GYTS 2000 -2009)

	Unmatched	Matched
VARIABLES	Odds ratio	Odds ratio
Affordability (RIP)	0.981**	0.974***
	(0.009)	(0.009)
Offered free cigarette (Ref=no)	1.491***	0.517***
	(0.216)	(0.071)
Sex (Ref = male)	0.599***	0.615***
	(0.072)	(0.076)
At least one parent smokes (Ref =no)	2.131***	0.862
	(0.280)	(0.104)
Family/class discussion (Ref = no)	1.001	1.711***
	(0.133)	(0.230)
At least one friend smokes (Ref=no)	4.109***	1.094
	(0.520)	(0.126)
Exposure to adverts (Ref=no)	1.155	1.027
	(0.140)	(0.121)
Age	1.150***	0.991
	(0.042)	(0.031)
Heard of anti-smoking message/campaign (Ref=no)	1.342*	2.048***
	(0.217)	(0.321)
Survey cycle (ref=2000)		
2006	0.958	0.880
	(0.146)	(0.138)
2009	1.108	1.003
	(0.171)	(0.159)
Log (time)	1.110	1.393***
	(0.106)	(0.146)
Constant	0.000***	0.048***
	(0.000)	(0.024)
Observations	106,673	10,078
Number of people	15,201	1,611

For peer review only

Ever-smokers	611	611
Pseudo R-squared	0.0815	0.0448
Chi2	439.2***	91.84***

Robust standard errors in parentheses
*** p<0.01, ** p<0.05, * p<0.1

Table 3: **Effect of RIP on smoking initiation among adolescents** (GYTS 2017)

	Unmatched	Matched
VARIABLES	Odds ratio	Odds ratio
Affordability (RIP)	0.821**	0.804***
	(0.066)	(0.065)
Sex (Ref=male)	0.659**	0.902
	(0.120)	(0.177)
Offered free cigarettes (Ref= no)	3.403***	0.978
	(0.726)	(0.221)
Heard of anti-smoking message (Ref=no)	1.165	1.009
	(0.213)	(0.192)
Exposed to tobacco adverts (Ref= no)	3.030***	1.893***
	(0.622)	(0.421)
Smoke free policies awareness (Ref=no)	1.250	1.160
	(0.329)	(0.332)
Age	1.847***	1.793***
	(0.294)	(0.279)
Class discussion on tobacco harms (Ref=no)	0.795	1.278
	(0.138)	(0.241)
Log(time)	0.104***	0.119***
	(0.067)	(0.076)
Constant	0.000***	0.002***
	(0.000)	(0.002)
Observations	37,654	4,850
Number of people	5,301	747
Ever-smokers	231	206
Pseudo R-squared	0.0599	0.0292
Chi2	158***	38.72***

Robust standard errors in parentheses
*** p<0.01, ** p<0.05, * p<0.1

For peer review only - http://bmjopen.bmj.com/site/about/guidelines.xhtml

Similarly, in Table 3, in the unmatched sample, an adolescent faced with a unit increase in RIP has about 18% (i.e., 1 – 0.821) × 100 = 17.9%)) lower probability of starting smoking than his/her counterpart who is not subjected to the same increase.

Other significant factors that influence smoking initiation in our two samples include whether the adolescent's parents (OR = 2.131, p<0.01) or friends (OR = 4.109, p<0.01) smoke. In addition, adolescents who are offered free cigarettes have a high probability of initiating smoking (OR = 1.491, p<0.01) compared to those who receive no such offer for the 2000 - 2009 wave (Table 2). In the 2017 wave, the odds of adolescents starting smoking when given cigarettes freely is 3.403 (p<0.01) (Table 3).

In the matched sample (Table 2), 611 ever-smokers were matched to their two nearest neighbors (1000 never-smokers) which produced a sample of 1,611 adolescents with similar characteristics. An adolescent subjected to a unit increase in RIP has a 0.97 times lower chance of initiating smoking (OR = 0.974, p<0.01) compared to those not exposed to the same increase in RIP. Similarly in the matched sample of the GYTS 2017 (Table 3), a unit increase in RIP is associated with 0.80 times lower odds of smoking initiation (OR = 0.804, p<0.01).

Getting free cigarettes (OR = 0.517, p<0.01), family/class discussion on tobacco (OR = 1.711, p<0.01), and hearing antismoking messages (OR = 2.048, p<0.01) are all found to be statistically significant in influencing smoking initiation in the matched sample (Table 2). However, the odds for these variables are contrary to *a priori* expectations. Similarly, in the matched sample (Table 2), the likelihood of initiating smoking among females is lower. The results imply that females have about 39% lower probability of initiating smoking (OR = 0.615, p<0.01) than males.

3.3. Affordability Elasticities

For peer review only - http://bmjopen.bmj.com/site/about/guidelines.xhtml

In the unmatched sample, the estimated affordability elasticity is -0.372 [CI: -0.701 to -0.042] for the 2000-2009 sample and -1.247 [-2.248 to -0.246] for the 2017 sample. These elasticities are statistically significant at the 5% level. By sex, the affordability elasticity is -0.137 for males and -0.888 for females for the 2000-2009 sample, but only that of females is statistically significant (p<0.01). The elasticities are higher for 2017 GYTS (-0.938 for males and -1.610 for females). The results are presented in Table 4.

Table 4: Affordability Elasticity estimates

	Both sexes		Male		Female	
Panel A: 2000 -2009						
	Percentage changes (d(lny)/d(lnx))					
VARIABLES	Unmatched	Matched	Unmatched	Matched	Unmatched	Matched
Affordability	-0.372**	-0.490***	-0.137	-0.326	-0.888***	-0.928***
	(0.168)	(0.168)	(0.219)	(0.216)	(0.253)	(0.258)
95% CI	-0.701 to -0.042	-0.818 to -0.161	-0.567 to 0.292	-0.749 to 0.097	-1.384 to -0.392	-1.434 to -0.422
Observations	106,673	10,078	55,396	5,648	51,277	4,430
Panel B: 2017 GYTS						
Affordability	-1.247**	-1.349***	-0.938**	-1.045**	-1.610*	1.518**
	(0.511)	(0.500)	(0.474)	(0.484)	(0.866)	(0.778)
95% CI	-2.248 to -0.246	-2.328 to - 0.369	-1.867 to -0.008	-1.993 to - 0.096	-3.307 to - 0.087	-3.043 to - 0.007
Observations	37,654	4,850	18,084	2,807	19,570	2,043

Standard errors in parentheses
*** p<0.01, ** p<0.05, * p<0.1

In the matched sample for the 2000-2009 GYTS, the overall elasticity is -0.490 [CI: -0.818 to -0.161] for both sexes (Table 4), which is similar to that of the unmatched sample. For males, the effect of changes in RIP is statistically insignificant. Among females, a percentage increase in RIP is associated with a 0.928% lower probability of smoking initiation. The elasticities for both males and females in the 2017 GYTS were negative, statistically significant, and more than unity.

For peer review only

For peer review only - http://bmjopen.bmj.com/site/about/guidelines.xhtml

The mean and median standardized difference for the covariates used in matching show that the matching satisfies the balancing test (results not reported). The mean and median standardized difference is 2.3%, which is below the normal 10% threshold. Therefore, the balancing property is satisfied.

5. DISCUSSION AND CONCLUSION

In this study, increasing the RIP of cigarettes is significantly associated with a lower probability of initiating smoking. This finding is consistent with international literature, including the few existing studies in SSA that have reported that making cigarettes less affordable lowers the likelihood of smoking initiation among young people.[17 18 26] In addition, the results from the unmatched 2000-2009 sample suggest that males are not responsive to changes in RIP whilst females are. Nevertheless, in the matched sample analysis, especially using the 2017 GYTS, both males and females are responsive to changes in RIP. Indeed, the issue of affordability becomes more important given that Ghana's per capita income has been growing at an average of 4.4% annually in the last decade.[30]

Parental smoking increased the odds of smoking initiating. This points to the parental influence on the lifestyle of adolescents. Adolescents whose parents smoke may perceive smoking as acceptable behavior. Previous studies have reported similar findings.[10 27 31 53] The odds of smoking initiation are higher for those whose friends' smoke. This points to the influence of peers, and is consistent, for example, with Mak, et al. [31]. Those who were offered free cigarettes by sales representatives were more likely to initiate smoking. All forms of tobacco promotion and advertising were banned in Ghana in 1982. However, the tobacco industry seems to be breaking these laws, because 12.44% of youth reported being offered a cigarette for free. This observation, together with our results, suggest the need to strengthen

For peer review only - http://bmjopen.bmj.com/site/about/guidelines.xhtml

the enforcement of the existing ban on all forms of tobacco advertising and promotion in Ghana.

This study has several limitations. The GYTS is a self-reporting survey, which means the responses are prone to recall bias even in cases where the adolescents are required to answer questions about events that occurred in the past 30 days. For instance, students may not recall the exact age at which they tried their first puff. There is also a social desirability bias when self-reporting behaviors such as smoking, especially among females. The weakness of our measure of smoking initiation is that it may not predict regular smoking behavior well.[42 43] In addition, there are other important factors affecting smoking uptake among the youth that are not included in this study. For instance, changing community norms regarding smoking, the enforcement of laws regarding the sale of cigarettes to minors, and changes in the social image of smoking are key factors that may influence smoking participation,[54 55] but are not included in the models estimated.

In conclusion, making cigarettes less affordable is associated with a lower probability of smoking initiation among adolescents in Ghana. This supports the use of price measures, through higher excise taxes, as effective strategies to decrease smoking initiation among adolescents in Ghana. Since incomes are rising at the average of 4.4% annually[30], tobacco taxes need to be adjusted regularly to ensure that cigarettes or other tobacco products become less affordable over time in order to discourage young people from initiating smoking and to encourage smokers to quit.

Author Contributions: Conceptualization, M.K.B.; methodology, M.K.B; software, M.K.B.; validation, M.K.B.., M.I., E.N.T., N.D.M., and H.R.; formal analysis, M.K.B.; investigation, M.K.B., M.I., E.N.T., N.D.M., and H.R.; data curation, M.K.B; writing—original draft preparation, M.K.B., M.I., E.N.T., N.D.M., and H.R.; writing—review and editing, M.K.B., M.I., E.N.T., N.D.M., H.R.; project administration, M.K.B.; supervision, H.R.; funding acquisition, H.R. All authors have read and approved the manuscript.

For peer review only - http://bmjopen.bmj.com/site/about/guidelines.xhtml

Funding: Funding for this study was provided by Cancer Research UK (Grant number: C62640/A24723) and International Development Research Centre (Grant Number: 108820-001) with additional support from the SAMRC/Wits Centre for Health Economics and Decision Science – PRICELESS SA (grant number 23108). Funding for ENT and NDM is acknowledged from the Tobacco Control Capacity Programme (Grant Number: MR/P027946/2) supported by UK Research and Innovation (UKRI) with funding from the Global Challenges Research Fund (GCRF). The funders had no role in study design, data collection and analysis, or preparation of the manuscript.

Conflicts of Interest: The authors declare that they have no conflict of interest.

Research Ethics Approval: Human Participants

This study does not involve human participants.

Research Ethics Approval: Animals

This study does not involve animal subjects.

Patient consent for publication: No patient involved.

Data Availability statement: The publicly available data can be accessed: https://nccd.cdc.gov/GTSSDataSurveyResources/Ancillary/DataReports.aspx?CAID=2. The relative income price data are available from the authors on reasonable request.

REFERENCES

1. Doll R, Peto R, Boreham J, et al. Mortality in relation to smoking: 50 years' observations on male British doctors. *Bmj* 2004;328(7455):1519.

2. Winkler V, Mangolo NJ, Becher H. Lung cancer in South Africa: a forecast to 2025 based on smoking prevalence data. *BMJ open* 2015;5(3):e006993.

For peer review only - http://bmjopen.bmj.com/site/about/guidelines.xhtml

3. World Health Organization. Tobacco: Key Facts. 2020 [updated 27 May 2020. Available from: https://www.who.int/news-room/fact-sheets/detail/tobacco accessed 22 November 2020.

4. Goodchild M, Nargis N, d'Espaignet ET. Global economic cost of smoking-attributable diseases. *Tobacco Control* 2018;27(1):58-64.

5. Boachie MK, Rossouw L, Ross H. The Economic Cost of Smoking in South Africa, 2016. *Nicotine Tob Res* 2021;23(2):286 - 93. doi: 10.1093/ntr/ntaa162

6. USDHHS. Preventing tobacco use among youth and young adults: A report of the surgeon general 2012 [Available from: http://www.surgeongeneral.gov/library/reports/preventing-youth-tobacco-use/full-report.pdf (accessed: Jan 3, 2019).

7. World Health Organization. WHO global report on trends in prevalence of tobacco use 2000-2025. 3rd ed. Geneva: World Health Organization, 2019.

8. CDC, WHO, MOH. GLOBAL YOUTH TOBACCO SURVEY: Factsheet, Ghana 2017, 2018.

9. World Health Organization. WHO global report on trends in prevalence of tobacco smoking 2015. Geneva: World Health Organization, 2015.

10. Vellios N, Van Walbeek C. Determinants of regular smoking onset in South Africa using duration analysis. *BMJ Open* 2016;6(7):e011076.

11. IARC. Effectiveness of tax and price policies for tobacco control. In: Cancer. IAfRo, ed. USA: International Agency for Research on Cancer, 2011.

12. Boachie MK, Ross H. Determinants of smoking intensity in South Africa: evidence from township communities. *Preventive Medicine Reports* 2020;19:101099.

13. Van Walbeek C. A simulation model to predict the fiscal and public health impact of a change in cigarette excise taxes. *Tobacco control* 2010;19(1):31-36.

14. Yurekli A, de Beye J. Design and administer tobacco taxes. *World Bank economics of tobacco toolkit* 2001(4)

15. Van Walbeek C. Raising Additional Government Revenues in Ghana by Raising the Excise Tax on Tobacco and Alcohol Washington, D.C.: WBG Global Tobacco Control

For peer review only - http://bmjopen.bmj.com/site/about/guidelines.xhtml

Program, World Bank Group; 2014 [Available from: http://documents.worldbank.org/curated/en/133561561707742558/pdf/Raising-Additional-Government-Revenues-in-Ghana-by-Raising-the-Excise-Tax-on-Tobacco-and-Alcohol.pdf accessed 15 July 2019.

16. He Y, Shang C, Chaloupka FJ. The association between cigarette affordability and consumption: An update. *PLOS ONE* 2018;13(12):e0200665. doi: 10.1371/journal.pone.0200665

17. Nargis N, Stoklosa M, Shang C, et al. Price, Income, and Affordability as the Determinants of Tobacco Consumption: A Practitioner's Guide to Tobacco Taxation. *Nicotine and Tobacco Research* 2021;23(1):40-47.

18. Blecher E, Van Walbeek C. An international analysis of cigarette affordability. *Tobacco Control* 2004;13(4):339-46.

19. Ding A. Youth are more sensitive to price changes in cigarettes than adults. *The Yale journal of biology and medicine* 2003;76(3):115.

20. Ho L-M, Schafferer C, Lee J-M, et al. The effect of cigarette price increases on cigarette consumption, tax revenue, and smoking-related death in Africa from 1999 to 2013. *International journal of public health* 2017;62(8):899-909.

21. Chelwa G, van Walbeek C. Does cigarette demand respond to price increases in Uganda? Price elasticity estimates using the Uganda National Panel Survey and Deaton's method. *BMJ open* 2019;9(3):e026150. doi: 10.1136/bmjopen-2018-026150 [published Online First: 2019/03/23]

22. Stoklosa M, Goma F, Nargis N, et al. Price, tax and tobacco product substitution in Zambia: findings from the ITC Zambia Surveys. *Tob Control* 2019;28(Suppl 1):s45-s52. doi: 10.1136/tobaccocontrol-2017-054037 [published Online First: 2018/03/27]

23. Mukong AK, Tingum EN. The demand for cigarettes: New evidence from South Africa. *Development Southern Africa* 2020;37(1):40-54.

24. Tingum EN, Mukong AK, Mdege N. The effects of price and non-price policies on cigarette consumption in South Africa. *Tob Induc Dis* 2020;18(July) doi: 10.18332/tid/123424

For peer review only - http://bmjopen.bmj.com/site/about/guidelines.xhtml

25. Kidane A, Mduma J, Naho A, et al. The Demand for Cigarettes in Tanzania and Implications for Tobacco Taxation Policy. *Advances in economics and business* 2015;3(10):428.

26. Kostova D. A (nearly) global look at the dynamics of youth smoking initiation and cessation: the role of cigarette prices. *Applied Economics* 2013;45(28):3943-51.

27. Asare S, Stoklosa M, Drope J, et al. Effects of Prices on Youth Cigarette Smoking and Tobacco Use Initiation in Ghana and Nigeria. *International Journal of Environmental Research and Public Health* 2019;16(17):3114.

28. Ministry of Health. Needs assessment for the Implementation of WHO FCTC in Ghana. Accra: Ministry of Health,, 2010.

29. Gov't. of Ghana. Customs and Excise (Duties and Other Taxes) Amendment Act 739. , 2007.

30. World Bank. World Development Indicators.: World Bank; 2019 [Available from: https://databank.worldbank.org/source/world-development-indicators. accessed 01 Jan 2020.

31. Mak K-K, Ho S-Y, Day JR. Smoking of parents and best friend—independent and combined effects on adolescent smoking and intention to initiate and quit smoking. *Nicotine Tob Res* 2012;14(9):1057-64.

32. Guindon GE, Paraje GR, Chávez R. Prices, inflation, and smoking onset: the case of Argentina. *Economic Inquiry* 2018;56(1):424-45.

33. Dauchy E, Ross H. The Effect of Price and Tax Policies on the Decision to Smoke among Men in Kenya. *Addiction* 2019;114(7):1249-63.

34. Li M. Using the propensity score method to estimate causal effects: A review and practical guide. *Organizational Research Methods* 2013;16(2):188-226.

35. Warren CW. Tobacco use among youth: a cross country comparison. *Tobacco Control* 2002;11(3):252-70.

36. Boachie MK, Immurana M, Agyemang JK, et al. Cigarette prices and smoking experimentation in Sierra Leone: an exploratory study. *Tobacco Use Insights* 2022;forthcoming

For peer review only - http://bmjopen.bmj.com/site/about/guidelines.xhtml

37. Gov't. of Ghana. Public Health Act, 2012 Act 851. Ghana 2012.

38. Wellington E. Ghana Global Youth Tobacco Survey (GYTS), Factsheet. 2006 [Available from: http://www.afro.who.int/en/downloads/docdownload/1887-ghana-gyts-2006-factsheet.html (Retrieved Jan 28, 2019).

39. Cadmus EO, Ayo-Yusuf OA. The effect of smokeless tobacco use and exposure to cigarette promotions on smoking intention among youths in Ghana. *Cogent Medicine* 2018;5(1):1531459.

40. CDC and WHO. Ghana global youth tobacco survey. Atlanta, United States: Centers for Disease Control and Prevention (CDC). 2006.

41. Freedman KS, Nelson NM, Feldman LL. Smoking initiation among young adults in the United States and Canada, 1998-2010: a systematic review. *Preventing chronic disease* 2012;9

42. Azagba S, Baskerville NB, Minaker L. A comparison of adolescent smoking initiation measures on predicting future smoking behavior. *Preventive medicine reports* 2015;2:174-77.

43. Reidpath DD, Davey TM, Kadirvelu A, et al. Does one cigarette make an adolescent smoker, and is it influenced by age and age of smoking initiation? Evidence of association from the US Youth Risk Behavior Surveillance System (2011). *Preventive medicine* 2014;59:37-41.

44. Centers for Disease Control Prevention. Selected cigarette smoking initiation and quitting behaviors among high school students--United States, 1997. *MMWR Morbidity and mortality weekly report* 1998;47(19):386.

45. Blecher E. Targeting the affordability of cigarettes: a new benchmark for taxation policy in low-income and-middle-income countries. *Tobacco control* 2010;19(4):325-30.

46. Nargis N, Stoklosa M, Drope J, et al. Trend in the affordability of tobacco products in Bangladesh: findings from the ITC Bangladesh Surveys. *Tobacco control* 2019;28(Suppl 1):s20-s30.

For peer review only

For peer review only - http://bmjopen.bmj.com/site/about/guidelines.xhtml

47. Philip Morris International. World summary of cigarette retail prices, tax burdens and tax incidence.: Philips Moris Records 1996 [Available from: https://www.industrydocuments.ucsf.edu/docs/#id=glhf0066.

48. Ghana Gto. Report card on the WHO Framework Convention on Tobacco Control., 2009.

49. Ghana Statistical Service. Consumer Price Index (CPI): Time Series In: Service GS, ed. Accra, 2012.

50. Austin PC, Jembere N, Chiu M. Propensity score matching and complex surveys. *Statistical methods in medical research* 2018;27(4):1240-57.

51. DuGoff EH, Schuler M, Stuart EA. Generalizing observational study results: applying propensity score methods to complex surveys. *Health services research* 2014;49(1):284-303.

52. Jenkins SP. Survival Analysis with Stata. [Available from: https://www.iser.essex.ac.uk/resources/survival-analysis-with-stata accessed 01 January 2020.

53. Gilman SE, Rende R, Boergers J, et al. Parental smoking and adolescent smoking initiation: an intergenerational perspective on tobacco control. *Pediatrics* 2009;123(2):e274-e81.

54. Gilpin EA, Pierce JP. Trends in adolescent smoking initiation in the United States: is tobacco marketing an influence? *Tobacco Control* 1997;6(2):122-27.

55. Cawley J, Markowitz S, Tauras J. Obesity, cigarette prices, youth access laws and adolescent smoking initiation. *Eastern Economic Journal* 2006;32(1):149-70.

For peer review only

For peer review only - http://bmjopen.bmj.com/site/about/guidelines.xhtml

STROBE Statement—Checklist of items that should be included in reports of ***cross-sectional studies***

	Item No	Recommendation	Page No
**Title and abstract**	1	(*a*) Indicate the study's design with a commonly used term in the title or the abstract	1
		(*b*) Provide in the abstract an informative and balanced summary of what was done and what was found	2
**Introduction**			
Background/rationale	2	Explain the scientific background and rationale for the investigation being reported	5-7
Objectives	3	State specific objectives, including any prespecified hypotheses	6
**Methods**			
Study design	4	Present key elements of study design early in the paper	7
Setting	5	Describe the setting, locations, and relevant dates, including periods of recruitment, exposure, follow-up, and data collection	7-8
Participants	6	(*a*) Give the eligibility criteria, and the sources and methods of selection of participants	7-9
Variables	7	Clearly define all outcomes, exposures, predictors, potential confounders, and effect modifiers. Give diagnostic criteria, if applicable	8-9
Data sources/ measurement	8*	For each variable of interest, give sources of data and details of methods of assessment (measurement). Describe comparability of assessment methods if there is more than one group	8-9
Bias	9	Describe any efforts to address potential sources of bias	10
Study size	10	Explain how the study size was arrived at	8
Quantitative variables	11	Explain how quantitative variables were handled in the analyses. If applicable, describe which groupings were chosen and why	9
Statistical methods	12	(*a*) Describe all statistical methods, including those used to control for confounding	9-10
		(*b*) Describe any methods used to examine subgroups and interactions	9-10
		(*c*) Explain how missing data were addressed	9-10
		(*d*) If applicable, describe analytical methods taking account of sampling strategy	9-10
		(*e*) Describe any sensitivity analyses	11
**Results**			
Participants	13*	(a) Report numbers of individuals at each stage of study—eg numbers potentially eligible, examined for eligibility, confirmed eligible, included in the study, completing follow-up, and analysed	12
		(b) Give reasons for non-participation at each stage	
		(c) Consider use of a flow diagram	
Descriptive data	14*	(a) Give characteristics of study participants (eg demographic, clinical, social) and information on exposures and potential confounders	13
		(b) Indicate number of participants with missing data for each variable of interest	
Outcome data	15*	Report numbers of outcome events or summary measures	
Main results	16	(*a*) Give unadjusted estimates and, if applicable, confounder-adjusted estimates and their precision (eg, 95% confidence interval). Make clear which confounders were adjusted for and why they were included	13-17

For peer review only

For peer review only - http://bmjopen.bmj.com/site/about/guidelines.xhtml

		(*b*) Report category boundaries when continuous variables were categorized	
		(*c*) If relevant, consider translating estimates of relative risk into absolute risk for a meaningful time period	
Other analyses	17	Report other analyses done—eg analyses of subgroups and interactions, and sensitivity analyses	
**Discussion**			
Key results	18	Summarise key results with reference to study objectives	18-19
Limitations	19	Discuss limitations of the study, taking into account sources of potential bias or imprecision. Discuss both direction and magnitude of any potential bias	19
Interpretation	20	Give a cautious overall interpretation of results considering objectives, limitations, multiplicity of analyses, results from similar studies, and other relevant evidence	11-12
Generalisability	21	Discuss the generalisability (external validity) of the study results	19
**Other information**			
Funding	22	Give the source of funding and the role of the funders for the present study and, if applicable, for the original study on which the present article is based	20

*Give information separately for exposed and unexposed groups.

Note: An Explanation and Elaboration article discusses each checklist item and gives methodological background and published examples of transparent reporting. The STROBE checklist is best used in conjunction with this article (freely available on the Web sites of PLoS Medicine at http://www.plosmedicine.org/, Annals of Internal Medicine at http://www.annals.org/, and Epidemiology at http://www.epidem.com/). Information on the STROBE Initiative is available at www.strobe-statement.org.

For peer review only
